# Unraveling the important role of comammox *Nitrospira* to nitrification in the coastal aquaculture system

**DOI:** 10.3389/fmicb.2024.1355859

**Published:** 2024-04-23

**Authors:** Xueqin Yang, Yongjie Wu, Longfei Shu, Hang Gu, Fei Liu, Jijuan Ding, Jiaxiong Zeng, Cheng Wang, Zhili He, Meiying Xu, Feifei Liu, Xiafei Zheng, Bo Wu

**Affiliations:** ^1^Southern Marine Science and Engineering Guangdong Laboratory (Zhuhai), Environmental Microbiomics Research Center, School of Environmental Science and Engineering, Sun Yat-sen University, Guangzhou, China; ^2^Ministry of Ecology and Environment, South China Institute of Environmental Sciences, Guangzhou, China; ^3^College of Agronomy, Hunan Agricultural University, Changsha, China; ^4^State Key Laboratory of Applied Microbiology Southern China, Guangdong Institute of Microbiology, Guangdong Academy of Sciences, Guangzhou, China; ^5^Ninghai Institute of Mariculture Breeding and Seed Industry, Zhejiang Wanli University, Ningbo, China

**Keywords:** nitrogen addition, nitrifying community, comammox *Nitrospira*, network, contribution to nitrification

## Abstract

Increasing nitrogen (N) input to coastal ecosystems poses a serious environmental threat. It is important to understand the responses and feedback of N removal microbial communities, particularly nitrifiers including the newly recognized complete ammonia-oxidizers (comammox), to improve aquaculture sustainability. In this study, we conducted a holistic evaluation of the functional communities responsible for nitrification by quantifying and sequencing the key functional genes of comammox Nitrospira-amoA, AOA-amoA, AOB-amoA and Nitrospira-nxrB in fish ponds with different fish feeding levels and evaluated the contribution of nitrifiers in the nitrification process through experiments of mixing pure cultures. We found that higher fish feeding dramatically increased N-related concentration, affecting the nitrifying communities. Compared to AOA and AOB, comammox Nitrospira and NOB were more sensitive to environmental changes. Unexpectedly, we detected an equivalent abundance of comammox Nitrospira and AOB and observed an increase in the proportion of clade A in comammox Nitrospira with the increase in fish feeding. Furthermore, a simplified network and shift of keystone species from NOB to comammox Nitrospira were observed in higher fish-feeding ponds. Random forest analysis suggested that the comammox Nitrospira community played a critical role in the nitrification of eutrophic aquaculture ponds (40–70 μM). Through the additional experiment of mixing nitrifying pure cultures, we found that comammox Nitrospira is the primary contributor to the nitrification process at 200 μM ammonium. These results advance our understanding of nitrifying communities and highlight the importance of comammox Nitrospira in driving nitrification in eutrophic aquaculture systems.

## 1 Introduction

The microbially driven nitrification process, which converts ammonium to nitrate via nitrite, is a vital process of the N cycle. It is also the rate-limiting step for the N removal process (Daims et al., 2015; Kuypers et al., 2018). Newly enriched and further isolated bacteria (*Nitrospira inopinata*) were shown to possess the ability of complete ammonium oxidization to nitrate by one organism (Daims et al., 2015; van Kessel et al., 2015; Santoro, 2016; Kits et al., 2017). This type of bacteria was named complete ammonia-oxidizing (comammox) bacteria and was theoretically predicated by kinetic modeling (Costa et al., 2006). Before this, nitrification was considered a two-step process for more than one century, which was carried out by ammonia-oxidizing bacteria (AOB) or archaea (AOA) (Könneke et al., 2005) and nitrite-oxidizing bacteria (NOB), respectively (Daims et al., 2016). However, the diversity, composition, and interactions of these nitrifiers, especially the newly discovered comammox, in response to environmental changes, as well as their contribution to the nitrification process in nutrient-enriched aquaculture systems, are not yet fully understood.

The discovery of comammox raised researchers’ interests on their diversity, distribution, and composition as well as their contribution to the nitrification process. To the best of our knowledge, the phylogenetic analysis showed that all known comammox bacteria were annotated as *Nitrospira* sublineage II (Daims et al., 2015; van Kessel et al., 2015; Daims et al., 2016) and were further divided into two clades, namely comammox *Nitrospira* clades A and clades B (Lawson and Lucker, 2018; Xia et al., 2018). The only isolate of comammox bacteria (*N. inopinata*) was the representative strain of clades A, which was reported to have the highest ammonia affinity than that of most AOA and AOB except the pure culture of marine AOA (*Nitrosopumilus maritimus*) (Hu and He, 2017; Kits et al., 2017). Surprisingly, no comammox *Nitrospira* have been found in the open ocean (Zhu et al., 2022). Evidence from the target enrichment of comammox *Nitrospira* in a membrane bioreactor with limiting ammonium and their predominance in biofilms of rotating biological contactor (approximately 15 μM ammonium) (Spasov et al., 2020) and rapid sand filters of drinking water treatment plants (Palomo et al., 2016; Fowler et al., 2018) further confirmed the importance of comammox *Nitrospira* to the nitrification process in those oligotrophic ecosystems.

However, a growing body of environmental research found that comammox *Nitrospira* were also widely distributed in less oligotrophic or eutrophic environments, including coastal systems (Xia et al., 2018), agricultural soils (Orellana et al., 2018), and wastewater treatments (Daims et al., 2016; Pinto et al., 2016; Bartelme et al., 2017; Wang et al., 2017; Annavajhala et al., 2018; Fowler et al., 2018). For instance, a recent study showed the abundance of comammox *Nitrospira* was much higher than canonical nitrifiers in a eutrophic lake (Chaohu lake, NH_4_^+^ was as high as 10 mM) and their abundance was positively related to ammonium in the paddy soils of Shaoguan and Antu (Shi et al., 2020). These results suggested that comammox *Nitrospira* may play a crucial role in nutrient-enriched environments. A previous study showed that comammox *Nitrospira* had a higher diversity than AOA or AOB (Shi et al., 2020; Spasov et al., 2020). Furthermore, comammox *Nitrospira* clade A was suggested to have a wider distribution and greater abundance compared to clade B (Fowler et al., 2018; Xia et al., 2018; Xu et al., 2020). Compared to canonical nitrifiers, clade A comammox bacteria *Ca. N. nitrificans* were positively linked to other species and served as the keystone species (Shi et al., 2020). These results suggested the diversity and niche heterogeneity of comammox *Nitrospira* may explain their ubiquity in eutrophic conditions. Due to the essential roles of nitrifiers and the hitherto under-researched role of the newly discovered comammox *Nitrospira*, it is necessary to consider all nitrifiers and a combination of their community traits (diversity, composition, interaction network, and keystone species) to better comprehend the nitrification process in eutrophic ecosystems.

In our study, we investigated the diversity and composition of the nitrifiers in aquatic ponds which were nitrogen (N) enriched environments, as N is added in the form of formulated feeds, whereas only 11–36% of the input N is converted to fish biomass (Hargreaves, 1998; Hu et al., 2012; Yuan et al., 2019). Thus, the remaining unabsorbed N which is converted into ammonium through ammonification enters into aquaculture ponds and adjacent ecosystems (Hargreaves, 1998; Hu et al., 2012; Yuan et al., 2019; Garlock et al., 2020). We selected 12 fish ponds with three replicates and divided them into two categories (small fish (SF) and large fish (LF) ponds) through different eutrophication degrees as LF ponds had higher eutrophication degrees with higher feeding amounts. We analyzed environmental variables, the abundance, composition, and diversity of comammox *Nitrospira amoA*, AOA *amoA*, AOB *amoA*, and *Nitrospira nxrB* to investigate how nitrifying communities, especially comammox *Nitrospira*, responded to different eutrophication levels. Furthermore, we used mix-culturing experiments of pure cultures (*N. inopinata*, *Nitrososphaera gargensis*, *Nitrosomonas communis,* and *N. moscoviensis*) with comparable ammonium addition to quantify their contribution to the nitrification process. We hypothesized that higher fish feeding altered the diversity, structure, and interactions of the nitrifying community, and comammox *Nitrospira* especially clade A played a crucial role in the nitrification process. This study advances our understanding of nitrifying communities, especially comammox *Nitrospira* in eutrophic conditions, and provides new insights into sustainable aquacultures.

## 2 Materials and methods

### 2.1 Site description and sampling

Aquaculture ponds were in Nansha, Guangzhou, China (22°60′97.88′′N, 113°62′18.05′′E) with each pond covering an area of 1.5 km^2^, and the depth of water was approximately 3 m. Water and sediment samples were collected with three biological replicates in 12 ponds for a total of 72 samples on 31 May 2018. A hydrophore sampler was used to collect surface water (1 L per sample, 50 cm depth below the surface), and a Van Veen grab sampler was used to collect sediment at a depth of approximately 0–8 cm (approximately 500 g). All the samples were collected in 1 day with clear weather. Ponds were divided into six small fish (SF) ponds and six large fish ponds (LF) according to the weight of grass carp per unit volume of the pond (0.03 kg/m^2^ vs. 0.06 kg/m^2^) in previous studies (Zhang et al., 2020; Zheng et al., 2021). The dosage of organic fish feed per m^2^ per day was approximately 5% of total fish weight. Samples were stored in a 4°C car refrigerator and transferred to the laboratory within 1 h at noon. Part of the fresh water and sediment samples were stored at 4°C for physicochemical analysis, and the rest were stored at −80°C for microbial DNA extraction.

### 2.2 Physicochemical analysis

Temperature, pH, DO, and salinity of the water (approximately 50 cm below the surface water) were measured *in situ* by portable meters, which were calibrated and used according to the manufacturer’s instructions (pH meter with temperature detector, SevenCompact™ pH Meter S210, Mettler-Toledo, United States; DO meter, 550A, YSI, USA; salinity meter, EUTECH SALT6+, Thermo Fisher Scientific, United States). The transparency of the water was measured *in situ* by Secchi disk. Chlorophyll a, total suspended solids (TSS), particle organic carbon (POC), dissolved organic carbon (DOC), and total organic carbon (TOC) of water were measured as previously described (Zhang et al., 2020; Zheng et al., 2021). Ammonium, nitrite, nitrate, total inorganic nitrogen (TIN), total organic nitrogen (TON), total nitrogen (TN), phosphate, total organic phosphorus (TOP), and total phosphorus (TP) were measured as previously described (Zheng et al., 2017a,b). The sediments were oven-dried at 65°C until a constant weight was reached, then sieved through a 200-mesh sieve to obtain sediment powder for the measurement of total carbon (sTC), nitrogen (sTN), and total sulfur (sTS) using an elemental analyzer (Vario TOC, Elemental, Germany). Total phosphorus (sTP), elemental sulfur (sES), and acid-volatile sulfur (sAVS) were determined as described previously (Zhang et al., 2020; Zheng et al., 2021).

### 2.3 Sediment microbial community DNA extraction

Microbial community DNA of 36 sediment samples (5 g of each sample) was extracted and purified by a combined protocol of the classic freeze–grind method and the DNeasy PowerSoil Kit (Qiagen, Dusseldorf, Germany) following the instructions in http://www.ou.edu/ieg/tools/protocols. The concentration and quality of extracted DNA were determined using a NanoDrop One spectrophotometer (Thermo Fisher Scientific, MA, United States) and stored at −20°C until subsequent experiments.

### 2.4 Quantitative real-time PCR analysis

The copy number of *amoA* genes from comammox *Nitrospira*, AOA and AOB, *nxrB* from *Nitrospira,* and 16S rRNA gene from bacterial communities were quantified by real-time quantitative PCR (qPCR) (Supplementary Table S1). Comammox *Nitrospira*, AOA and AOB *amoA*, *Nitrospira nxrB*, and 16S rRNA gene (V3 region) were obtained by using corresponding primer pairs (Supplementary Table S1) as primers and pure culture DNA as templates (*Nitrospira inopinata*, *Nitrososphaera gargensis*, *Nitrosomonas communis*, *Nitrospira moscoviensis,* and *E. coli*, respectively). The fragment of the V3 region was amplified using universal primers 338F and 536R (Xia et al., 2018). Purified PCR products were cloned into the pEASY TA vector (TransGen Biotech, Beijing, China), inserted into competent cells, and spread onto an LB plate. *E. coli* with target genes were selected to grow in a liquid LB medium, and then, plasmids were extracted as standards for quantification using the NucleoSpin Plasmid Kit (Macherey-Nagel, Düren, Germany). The concentration of plasmids was determined using Qubit 4.0 (Thermo Fisher Scientific, MA, United States). The qPCRs were run with three technical replicates in a Bio-Rad C1000 CFX96 real-time PCR system (United States). Each qPCR was prepared in a 20-μl reaction mix containing 10 μL of SYBR Green Supermix (Bio-Rad, United States), 4 μL of the suspension, 0.5 μL of each primer (10 μM), and 5 μL of ddH_2_O. The thermal qPCR program was as follows: 3 min at 98°C, followed by 40 cycles of 15 s at 98°C, 30s for annealing at 46, 55, 56, 52, and 57.5°C, respectively, for five marker genes, and 30s at 72°C, and then ended with a final extension at 72°C for 10 min. The amplification efficiency was between 90 and 110%, and the correlation coefficient (r^2^) of the standard curve was greater than 0.99.

### 2.5 PCR amplification, purification, and high-throughput sequencing

The functional maker genes of nitrifiers including comammox *Nitrospira amoA* (Ntsp-amoA 162F and Ntsp-amoA 359R) (Fowler et al., 2018), AOA *amoA* (Arch-amoA26F and Arch-amoA417R) (Park et al., 2008) and AOB *amoA* (amoA-1F and amoA-2R) (Rotthauwe et al., 1997), and *Nitrospira nxrB* (169Fand 638R) (Pester et al., 2014) were amplified using a PCR thermocycler instrument (T100, Bio-Rad, United States) (Supplementary Table S1). Although theoretically both NOB and known comammox contained *Nitrospira nxrB* gene (Annavajhala et al., 2018; Roots et al., 2019), here *Nitrospira nxrB* had approximately 10-fold higher abundance than comammox *Nitrospira amoA*. The amplification system with a total volume of 25 μL contained 12.5 μL of 2xEasyTaq SuperMix (TransGen Biotech, Beijing, China), 8.5 μL ddH_2_O, 1 μL of forward and reverse primers (10 μM), and 2 μL of appropriately diluted DNA (approximately 10–50 ng/μL). The thermal program was initial denaturation at 98°C for 3 min, followed by 25 cycles of denaturation at 98°C for 15 s, annealing for 30s (46, 56, 56, and 52°C for four marker genes, respectively), and extension at 72°C for 30s and ended with a final extension at 72°C for 10 min. PCR products were purified using an AxyPrep PCR Clean-up Kit (Axygen, MA, United States). The concentration of purified PCR product was determined using the Qubit 4.0 fluorometer (Thermo Fisher Scientific, MA, United States), and the quality was checked using a LabChip GX Touch HT nucleic acid analyzer (PerkinElmer, MA, United States). Purified PCR products after the quality control were pooled at equal concentrations for sequencing using an Illumina MiSeq-PE300 or NovaSeq-PE250 system according to the manufacturer’s guidelines. Raw sequence data have been submitted to NCBI Sequence Read Archives with an accession number PRJNA834749.

### 2.6 Sequencing data analysis

The raw data were split by their specific barcodes and then trimmed to remove sequencing primers. Sequence combination, adapter removal, length, and quality control were performed using Cutadapt (Martin, 2011) and fastp (Chen et al., 2018). Chimeras were removed through VSEARCH (Rognes et al., 2016) by mapping to the Ribosomal Database Project (RDP) database. RDP FrameBot was used to correct frameshift errors (Wang et al., 2013), and UNOISE3 was selected to generate the zero-radius operational taxonomic units (zOTUs) table (Edgar, 2013; Edgar, 2016). zOTUs table was filtered by discarding zOTUs with an average relative abundance of lower than 1/10,000. Finally, the total sequence number was resampled to 10,999. The reference database for annotation was downloaded from FunGene Pipeline.^1^ The quality before and after quality control was visualized by FastQC^2^ and multiQC.^3^

### 2.7 Molecular ecological network analysis of nitrifiers

Networks among nitrifiers in SF and LF pond sediments were constructed to elucidate their possible interactions by molecular ecological network analysis (MENA)^4^ (Deng et al., 2012) and visualized using gephi (version 0.9.2).^5^ zOTUs co-occurred in more than half of the samples that were kept for network construction via the random matrix theory (RMT) method. Network topological properties and module separation were further analyzed (Deng et al., 2012). The connectivity of each node was determined based on the within-module connectivity (Zi) and among-module connectivity (Pi) (Guimera and Amaral, 2005). Nodes were further divided into four categories (module hubs, network hubs, connectors, and peripherals) with a threshold of 2.5 (Zi) and 0.62 (Pi), respectively (Shi et al., 2016).

### 2.8 The relative contribution of comammox *Nitrospira*, AOA, and AOB to ammonium oxidation

According to the above sequencing results, four ubiquitous monocultures were selected to represent four types of nitrifiers with *N. inopinata* as comammox, *Nitrososphaera gargensis* as AOA, *Nitrosomonas communis* as AOB, and *N. moscoviensis* as NOB. Nitrifiers were cultivated in the medium as described previously (Daims et al., 2015) with 0.2, 1, and 2 mM ammonium at 37°C. The initial total biomass in different treatments was the same (4 × 10^4^ cells/mL). The measurement of cell density, ammonium, nitrite, and nitrate was described in our previous paper (Yang et al., 2022). Nitrate concentration was used when calculating the contribution of nitrifiers to nitrification as it is the product of nitrification. Three ammonium oxidizers were co-cultured with NOB separately [comammox+NOB (1), AOA + NOB (2), and AOB + NOB (3)]. Their maximum nitrate production rates were obtained by detecting the dynamics of nitrate concentration (μ1, μ2, and μ3). Similarly, four nitrifiers were equally mixed and co-cultured together (N4 communities), the maximum nitrate production rate (μ) and relative abundance of the nitrifiers at the endpoint were obtained (abundance of comammox *Nitrospira*: A_c_; abundance of AOA: A_A_; abundance of AOB: A_B_; and abundance of NOB: A_N_). The nitrate production rate of the N4 community in theory (μ_theory_) was roughly calculated as μ_theory_ = A_c_*μ_1_+ A_A_*μ_2_+ A_B_*μ_3_. There was no difference between μ_theory_ and μ which we obtained from the N4 community. The relative contribution of nitrifiers (W_c_, W_A_, and W_B_) in N4 to nitrification was calculated using maximum nitrate production rates of ammonium oxidizers as the weight of their relative abundance in the N4 community (e.g., W_c_ = A_c_ *μ_1_/μ_theory_*100%) (Brooks et al., 2015).

### 2.9 Statistical analysis

Alpha- and beta-diversity were calculated using Qiime (version 1.9.1) (Caporaso et al., 2010). Further analyses and figure plots including the alpha- and beta-diversity indices, principal coordinates analysis (PCoA), redundancy analysis (RDA), Mantel test, and random forest were performed using R (version 4.1.0) with different packages such as ggplot2 (Ginestet, 2011), vegan (Dixon, 2003), ieggr, rfPermute^6^ (Breiman, 2001) and ecodist (Goslee and Urban, 2007). The phylogenetic analysis was performed in MEGA X using the maximum-likelihood method (Kumar et al., 2018). Parametric and non-parametric tests were performed with IBM SPSS 22 (SPSS Inc., United States).

## 3 Results

### 3.1 Physicochemical properties in fish ponds

Compared to SF ponds, LF ponds had higher ammonium, nitrite, nitrate, total inorganic nitrogen (TIN), total nitrogen (TN), lower total organic nitrogen (TON), and pH with significant difference (*p* < 0.05). The differences in detailed physicochemical properties including basic water parameters, C, N, S, and P-related parameters in water and sediments of SF and LF ponds are summarized in Table 1. In total, 19 of 26 parameters showed significant (*p* < 0.05) differences between LF and SF ponds, indicating wide fluctuations of environmental conditions brought by higher N input to aquaculture ponds, and this may have an impact on the diversity and function of nitrifying communities.

**Table 1:** Physicochemical properties of water and sediment in SF and LF fish ponds, which are shown as mean ± SD (standard deviation) (*n* = 18).

	Parameters	SF ponds	LF ponds
Water	Temperature (°C)	32.86 ± 1.39^a^	33.68 ± 0.27^b^
	pH	8.34 ± 0.52^b^	7.91 ± 0.34^a^
	DO (mg/L)	8.81 ± 5.96	6.34 ± 2.51
	Salinity (%)	1.39 ± 0.34	1.50 ± 0.25
	Transparency (cm)	19.70 ± 6.65^b^	11.33 ± 1.81^a^
	Chla (μg/L)	135.6 ± 58.0^b^	88.19 ± 28.17^a^
	TSS (mg/L)	19.82 ± 19.94^a^	67.85 ± 13.35^b^
	POC (mg/L)	33.34 ± 14.68^b^	24.48 ± 2.70^a^
	DOC (mg/L)	31.26 ± 7.52^b^	15.60 ± 4.50^a^
	TOC (mg/L)	64.59 ± 17.43^b^	40.08 ± 4.56^a^
	NH_4_^+^ (mg/L)	0.554 ± 0.303	0.722 ± 0.382
	NO_2_^−^ (mg/L)	0.278 ± 0.317^a^	0.631 ± 0.365^b^
	NO_3_^−^ (mg/L)	1.11 ± 1.48^a^	4.48 ± 1.46^b^
	TIN (mg/L)	1.94 ± 1.72^a^	5.84 ± 1.87^b^
	TON (mg/L)	4.84 ± 1.03^b^	4.00 ± 0.84^a^
	TN (mg/L)	6.79 ± 2.00^a^	9.84 ± 1.35^b^
	PO_4_^−^ (mg/L)	0.397 ± 0.272^b^	0.085 ± 0.095^a^
	TOP (mg/L)	0.353 ± 0.210	0.240 ± 0.111
	TP (mg/L)	0.741 ± 0.392^b^	0.324 ± 0.065^a^
Sediment	sTC (%)	17.29 ± 6.56	17.20 ± 6.66
	sTOC (%)	11.73 ± 5.17	12.93 ± 5.80
	sTN (%)	1.51 ± 0.67	1.38 ± 0.62
	sTS (%)	2.82 ± 1.87^b^	0.99 ± 0.37^a^
	sES (mg/g)	20.23 ± 10.07^b^	7.65 ± 2.85^a^
	sAVS (mg/g)	437.3 ± 176.6^b^	126.0 ± 35.9^a^
	sTP (%)	0.142 ± 0.045^a^	0.184 ± 0.060^b^

Each parameter with different letters showed statistical differences (*p* < 0.05, *t*-test or Mann–Whitney U-test). *Fish ponds with different sizes of grass carp are abbreviated as SF and LF. Parameters determination from water included temperature, pH, dissolved oxygen (DO), salinity, transparency, chlorophyll a, total suspended solid (TSS), particle organic carbon (POC), dissolved organic carbon (DOC), total organic carbon (TOC), ammonium, nitrite, nitrate, total inorganic nitrogen (TIN), total organic nitrogen (TON), total nitrogen (TN), phosphate, total organic phosphorus (TOP), and total phosphorus (TP). Sediment properties included total carbon (sTC), total organic carbon (sTOC), total nitrogen (sTN), total sulfur (sTS), elemental sulfur (sES), acid-volatile sulfur (sAVS), and total phosphorus (sTP).

### 3.2 The abundance of comammox *Nitrospira* and AOB are of equal magnitude

**Figure 1:**
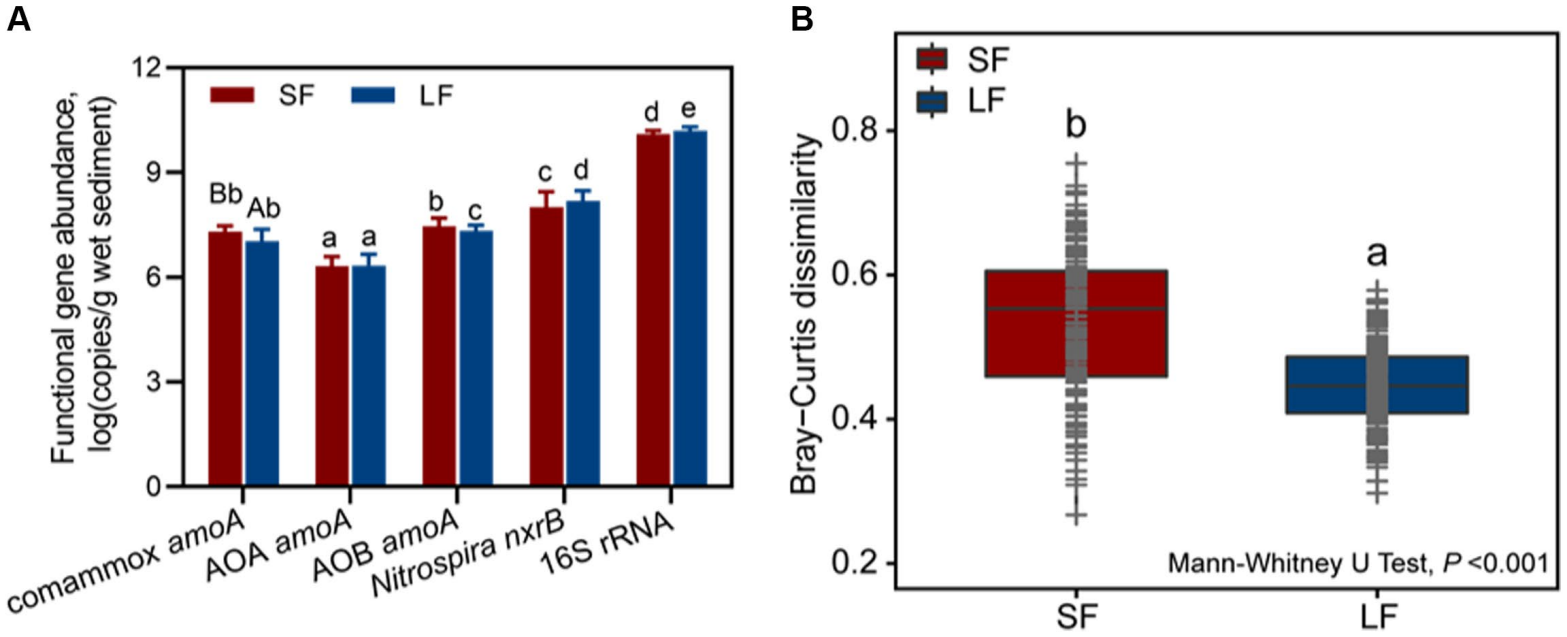
Abundance and beta diversity of nitrifiers in SF and LF ponds. **(A)** Abundances of comammox *Nitrospira amoA*, AOA *amoA*, AOB *amoA*, *Nitrospira nxrB,* and 16S rRNA genes in SF and LF pond sediments. Data are represented as mean ± SD (*n* = 18). Different capital letters mean a statistical significance (*p* < 0.05) between SF and LF pond sediments based on Student’s *t*-test; different small letters mean a statistical significance (*p* < 0.05) among different functional genes within the same fish size pond sediments based on one-way ANOVA. **(B)** Beta diversity of sediment nitrifying communities in the SF and LF ponds was estimated based on Bray–Curtis distance matrixes of all 36 samples. Different letters showed statistical differences (*p* < 0.001, Mann–Whitney *U*-test).

To determine the composition and predominance of sediment nitrifying communities in aquaculture ponds, we measured the abundance of comammox *Nitrospira amoA*, AOA *amoA*, AOB *amoA*, *Nitrospira nxrB,* and 16S rRNA genes by qPCR. No significant abundance difference was observed in the ratio of the total nitrifiers and total bacteria (16S rRNA) in different fish farming. In general, NOB were the most abundant nitrifiers in all fish ponds, followed by AOB, comammox *Nitrospira,* and AOA (Figure 1A). We found an unexpectedly high abundance of comammox *Nitrospira amoA* [(2.18 ± 0.23) × 10^7^-(1.37 ± 0.21) × 10^7^ copies/g wet sediment], which accounted for 39.44–32.23% of the ammonia oxidizer and 0.09–0.17%- of all the bacteria in the SF and LF ponds. The abundance of AOB *amoA* was from (2.31 ± 0.48) × 10^7^ to (2.30 ± 0.18) × 10^7^ copies/g wet sediment in the SF and LF ponds (Figure 1A and Supplementary Table S2). The abundance of comammox *Nitrospira amoA* in the SF ponds was significantly higher than in LF ponds (*p* < 0.05). The phylogenetic tree analysis revealed that sequences of comammox *Nitrospira amoA* (361 zOTUs) could be grouped into clade A (294 zOTUs) and clade B (67 zOTUs), and comammox *Nitrospira* clade A were the predominant clade in the SF (66.05%) and LF (89.41%) ponds (Supplementary Table S3).

### 3.3 The difference between comammox *Nitrospira* and other nitrifiers in community structure

**Figure 2:**
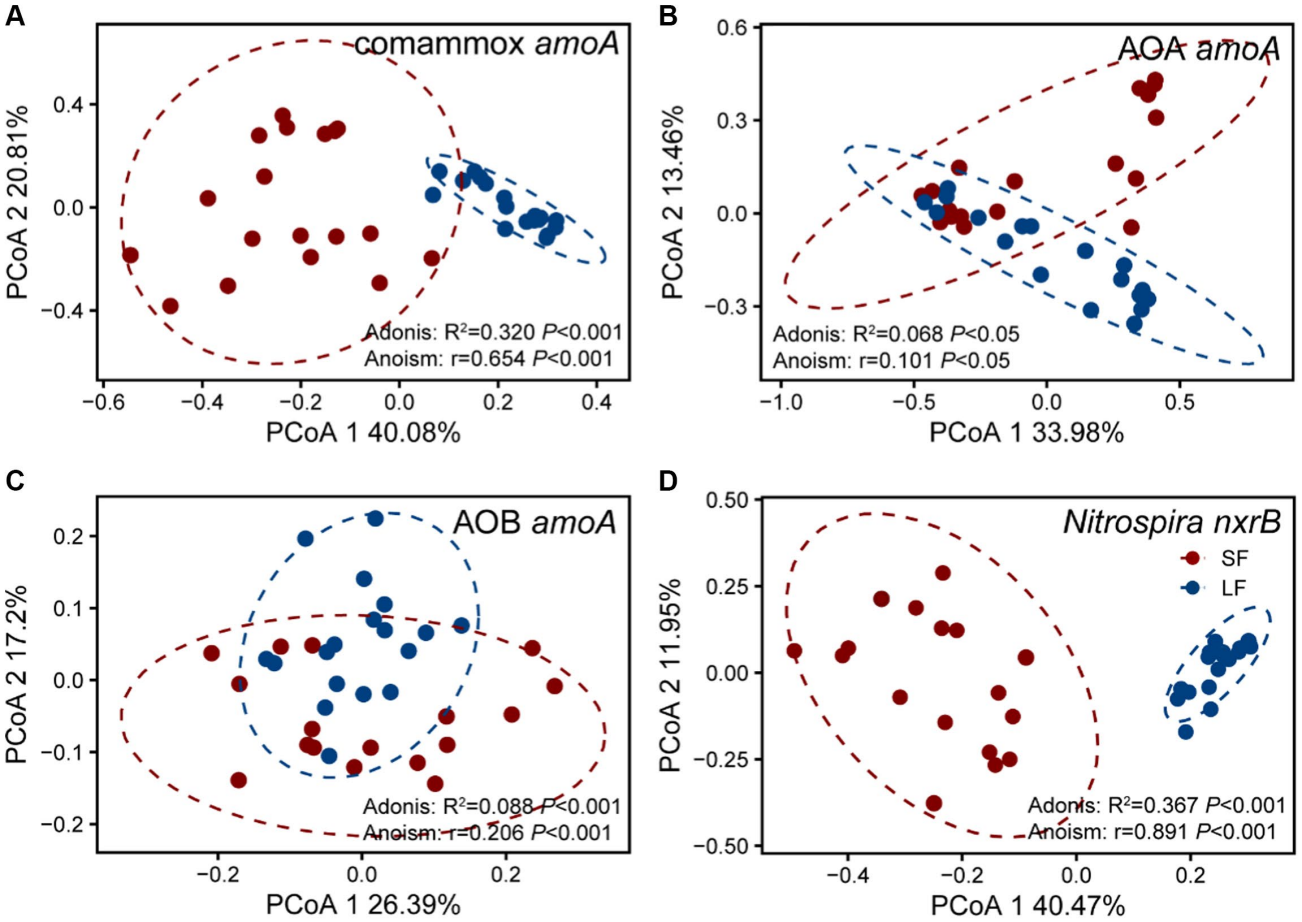
Principal co-ordinates analysis (PCoA) of four nitrifying communities in the SF and LF ponds, **(A)** AOA *amoA*, **(B)** AOB *amoA*, **(C)** Comammox *Nitrospira amoA*, and **(D)** *Nitrospira nxrB*. The samples in an ellipse showed 95% confidence within this group. The values of PCoA1 and PCoA2 labels were percentages of variations explained. Colors represent SF or LF pond sediments, respectively. The significance of dissimilarities was examined by the Adonis and Anosim tests.

To further understand the diversity and community structure of sediment nitrifying communities by fish farming, functional genes of comammox *Nitrospira amoA*, AOA *amoA*, AOB *amoA*, and *Nitrospira nxrB* were sequenced and analyzed. In general, α-diversity indices showed that NOB had higher diversity, followed by comammox *Nitrospira*, AOA, and AOB (Supplementary Table S4). Higher fish feeding mainly reduced the α-diversity of all nitrifying communities except comammox *Nitrospira*; they showed significantly (*p* < 0.05) higher values of observed richness and phylogenetic diversity in the LF ponds (Supplementary Table S4). For the within-beta diversity, LF pond sediments had a significantly (*p* < 0.001) lower value of the Bray–Curtis dissimilarity than that of SF pond sediments (Figure 1B; Supplementary Figure S1). In addition, the principal coordinates analysis (PCoA) revealed that higher dissimilarity of comammox *Nitrospira amoA* in the SF and LF ponds than AOA and AOB *amoA* (Figure 2), which together with the above results suggested that comammox *Nitrospira* may be more sensitive to aquaculture activities.

### 3.4 Comammox *Nitrospira* and NOB were more sensitive to environmental changes

**Figure 3:**
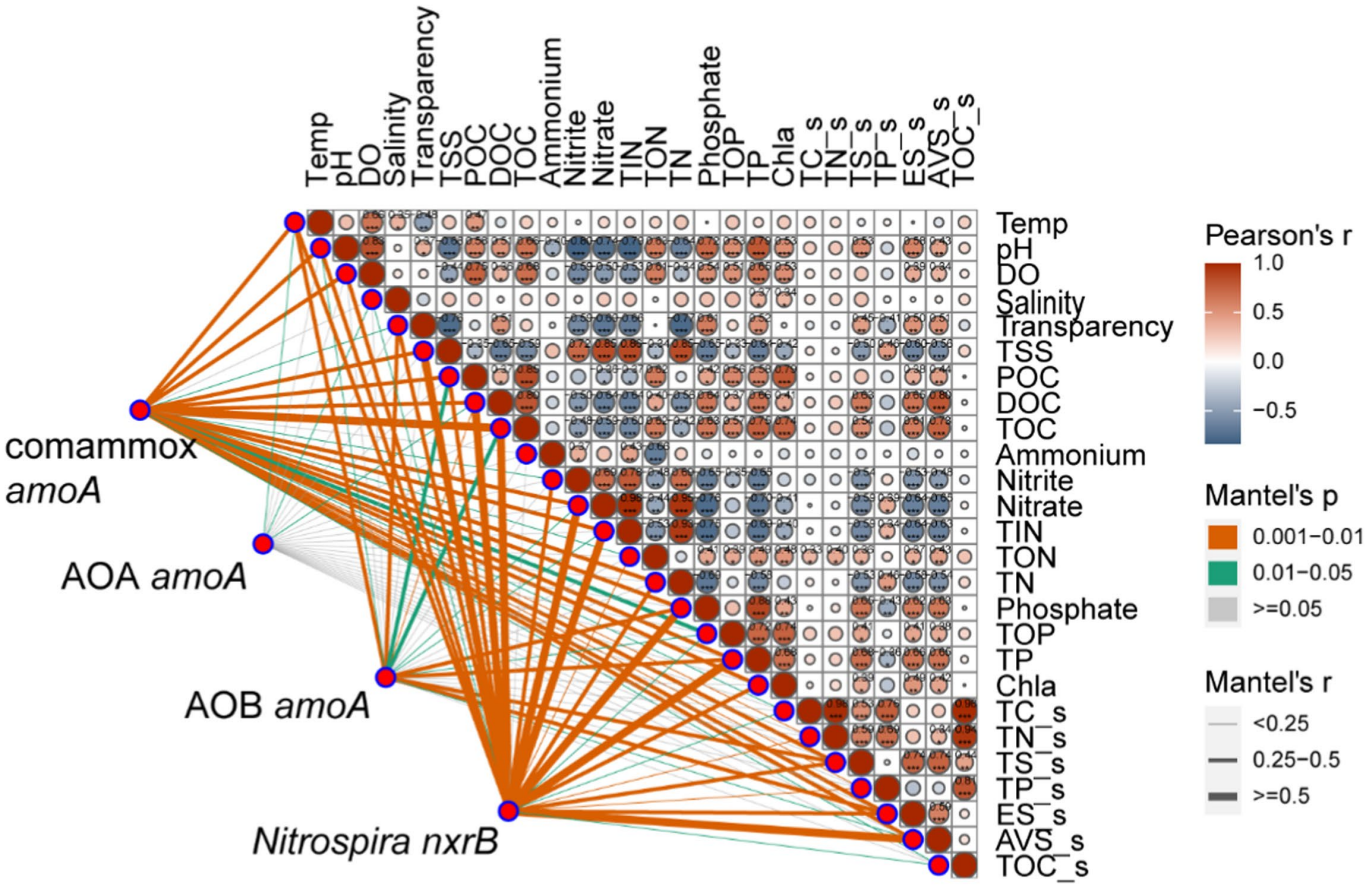
Relationships between nitrifying communities and environmental variables using the Mantel tests. The Mantel tests between nitrifiers (comammox *Nitrospira amoA*, AOA *amoA*, AOB *amoA,* and *Nitrospira nxrB*) and environmental variables in fish ponds based on Pearson’s correlations.

**Figure 4:**
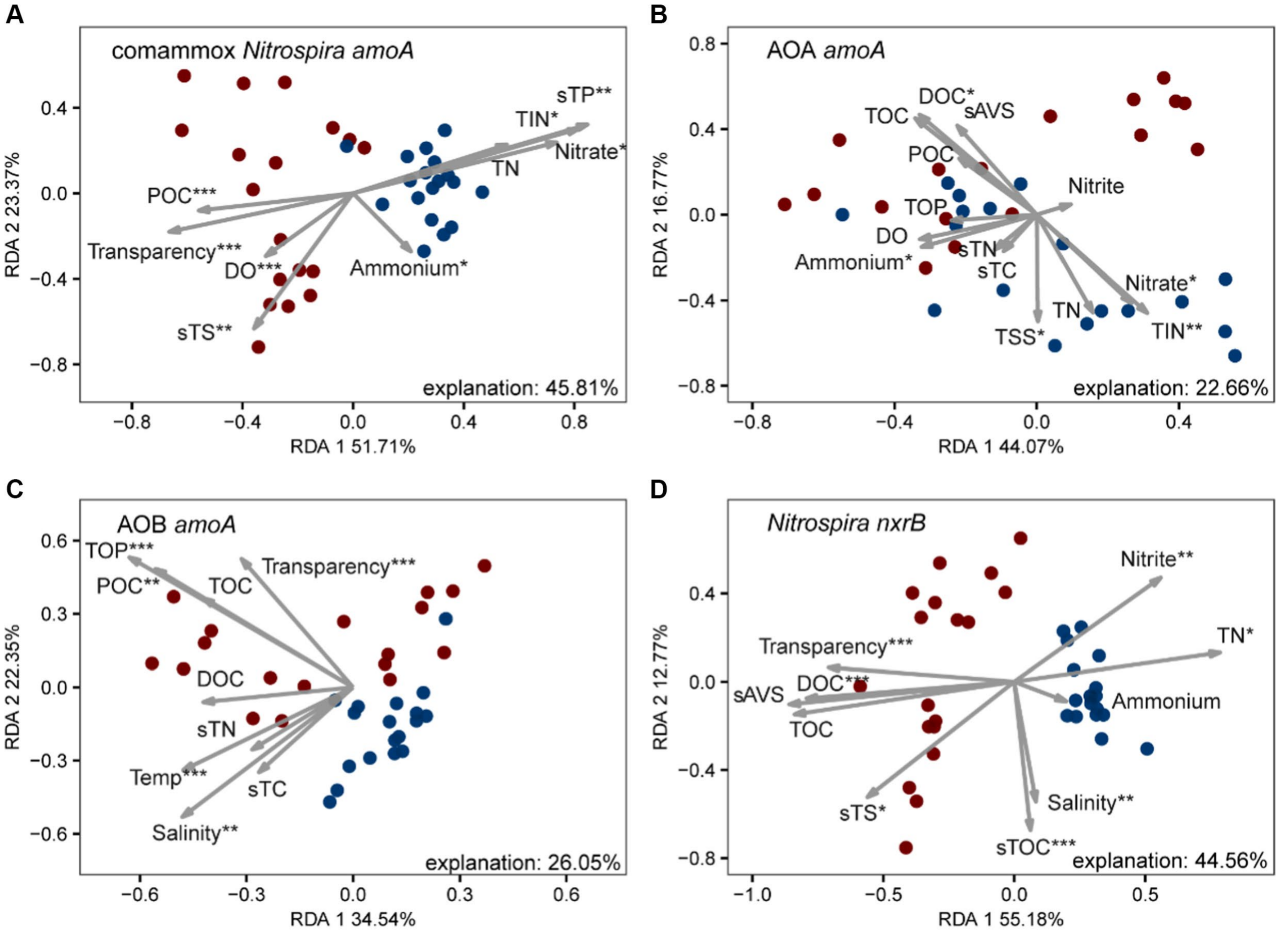
Redundancy analysis (RDA) of environmental drivers to nitrifying communities, **(A)** comammox *Nitrospira amoA*, **(B)** AOA *amoA*, **(C)** AOB *amoA*, and **(D)** *Nitrospira nxrB*. The values of RDA1 and RDA2 labels represented the percentages explained.

We further investigated the relative importance of environmental variables in the assembly of nitrifiers in aquaculture ponds. The Mantel tests showed that all 26 environmental variables were significantly (*p* < 0.05) correlated with the comammox *Nitrospira* and NOB community structure (except salinity for comammox *Nitrospira* and NH_4_^+^ in pond water for both) (Figure 3). TOC and TP were the most important drivers that shaped the composition of the comammox *Nitrospira*, while TSS and nitrate mainly affected the composition of NOB. AOA communities were only significantly (*p* < 0.05) related to temperature, salinity, and DOC. AOB communities were significantly (*p* < 0.05) related to 14 out of 26 environmental conditions including sES, phosphate, TP, sTS, POC, temperature, TOC, transparency, TOP, sAVS, pH, DOC, nitrate, and TIN (Figure 3). The RDA confirmed that these significant correlations and these environmental variables explained a large proportion of the variation in the comammox *Nitrospira* community (45.81%, *p* < 0.001) and the NOB community (44.56%, *p* < 0.001). However, the RDA model only explained 22.66% (*p* < 0.001) of variations in the AOA community and 26.05% (*p* < 0.001) of variations in the AOB community (Figure 4). The linear regression analysis also showed that the richness of comammox *Nitrospira* was positively correlated with temperature, DO, nitrate, and TIN (Supplementary Figure S2). Additionally, we analyzed the correlations between dominant nitrifiers and environmental variables. Relative abundances of the dominant comammox *Nitrospira* and NOB species were significantly (*p* < 0.05) correlated with N-related parameters especially nitrate, TIN, and TN (Supplementary Tables S5, S6). However, the dominant AOA and AOB species were relatively insensitive to related N parameters and other environmental changes compared to comammox *Nitrospira* and NOB species (Supplementary Tables S7, S8). These results indicated that comammox *Nitrospira* and NOB communities were more sensitive to environmental variables than AOA and AOB.

### 3.5 High fish feeding reduced networks of potential interactions

**Figure 5:**
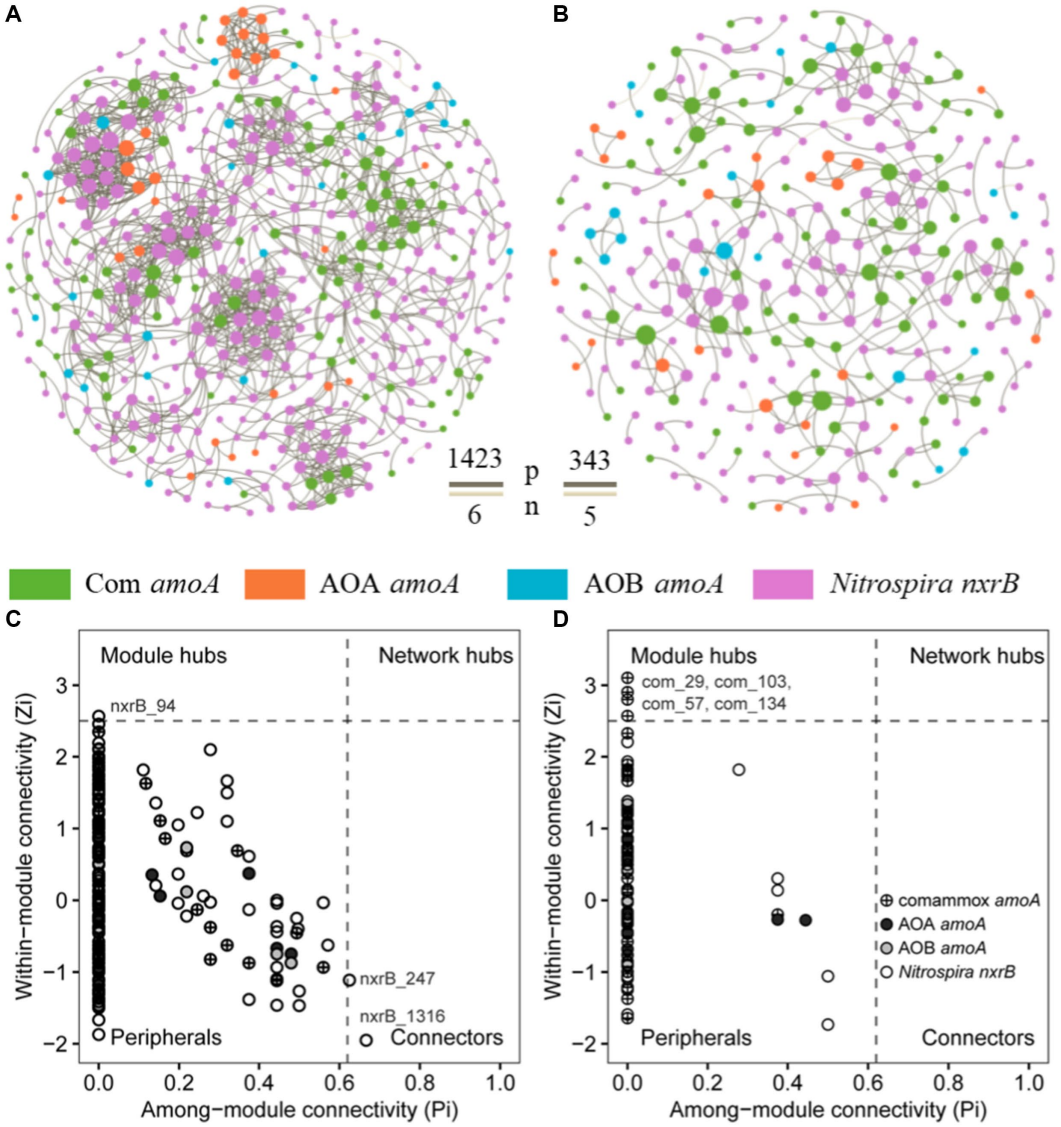
Co-occurrence networks of nitrifiers in aquaculture ponds, **(A)** SF ponds; **(B)** LF ponds, the brown and beige line between nodes showed positive and negative interactions, respectively. The network nodes in SF **(C)** and LF **(D)** ponds were separated by among-module connectivity (Pi) and within-module connectivity (Zi) within a threshold of 0.62 and 2.5 (Deng et al., 2012; Shi et al., 2016), respectively.

To explore the influence of fish farming on the interactions of nitrifiers in aquaculture systems, we constructed co-occurrence networks of sediment nitrifying communities in the SF and LF ponds (Figure 5; Supplementary Figure S2). Compared to the SF ponds, we found lower total nodes, links, average degree, density, connectedness, average path distance, and centralization of stress centrality of the co-occurrence network of nitrifying communities in LF ponds (Figures 5A,B; Supplementary Table S9). The potential interactions among nitrifiers were mostly positive (>98%) in both SF and LF ponds. In addition, we observed one module hub (nxrB_94) and two connectors (nxrB_247; nxrB_1316) all derived from *Nitrospira nxrB* in SF ponds and four module hubs (com_29, com_57, com_103, com_134) derived from comammox *Nitrospira amoA* in LF ponds (Figure 5; Supplementary Table S10). Among those core taxa, nxrB_94 was the most abundant zOTU in the SF ponds and its relative abundance was significantly (*p* < 0.05) higher in SF pond sediments than in LF ponds (0.40% vs. 0.02%). However, the abundance of com_29 in the LF pond samples was significantly (*p* < 0.05) higher than in the SF samples (0.08% vs. 1.07%). In addition, com_29 had the highest relative abundance than other keystones in LF pond sediments (Supplementary Table S10). Together, a less complex co-occurrence network of nitrifiers was observed in the LF ponds, and keystone species were shifted from *Nitrospira* nxrB species in the SF ponds to comammox *Nitrospira* in LF ponds in the coastal aquaculture ecosystem.

### 3.6 The contribution of comammox *Nitrospira* to nitrification is unneglectable in eutrophic conditions

**Figure 6:**
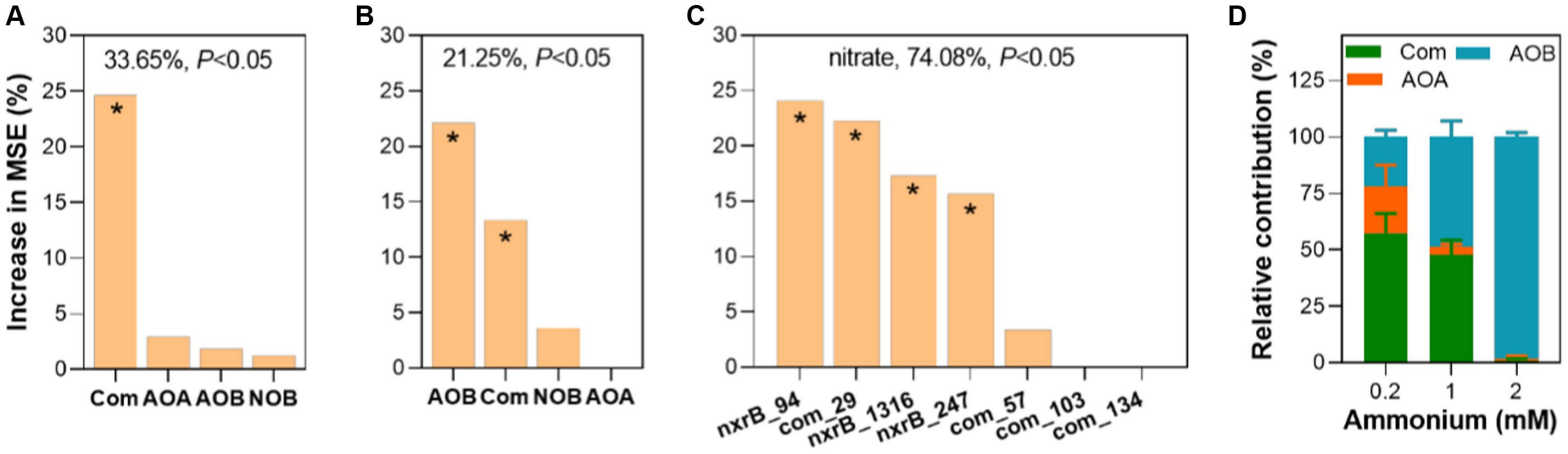
Exploring the contribution of four types of nitrifiers to nitrification. Contribution of the abundance **(A)** and richness **(B)** of four nitrifiers and keystone species **(C)** to nitrification potential analyzed by random forest. **(D)** The relative contribution of ammonium oxidizers to nitrification in synthetic nitrifying communities. The accuracy importance of measurement was computed for each tree and averaged over the forest (ntree = 1,000, nrep = 1,000). The overall explanatory degree and the significance of the models were calculated and shown. The higher importance of the predictor was reflected by a higher mean squared error (MSE). Asterisk (*) showed a statistically significant difference in the predictor (*p* < 0.05). More details can be found in the methods.

To estimate the potential contribution of nitrifiers to nitrification, the abundance and richness of nitrifiers and keystone species were used to predict nitrification potentials. We found that only the abundance of comammox *Nitrospira amoA* contributed to nitrification potential (24.67%) (Figure 6A) with a statistical significance (*p* < 0.05). However, both the richness of AOB *amoA* and comammox *Nitrospira amoA* contributed to nitrification potential with a statistical significance (*p* < 0.05) (Figure 6B). In addition, we assessed the contribution of core taxa to nitrification potential in fish ponds (Figure 6C). We found these core taxa explained a large proportion of nitrification potential variations. Among these core taxa, com_29 which belonged to comammox *Nitrospira* made a relatively important contribution to nitrification potential (*p* < 0.05). Moreover, we estimated the contribution of ammonium oxidizers to the nitrification process through mixed co-culture of four nitrifiers with three different ammonium supplies from 0.2 to 2 mM. We found that the relative contribution of comammox *Nitrospira* to nitrification was significantly higher than AOA and AOB under 0.2 mM ammonium (*p* < 0.05) (Figure 6D). When ammonium supply increased to 1 mM, the relative contribution of AOB and comammox was comparable (48.86% vs. 47.60%). Further increasing ammonium to 2 mM, AOB contributed to most of the nitrification (98.11%). These results confirmed the significant role of comammox *Nitrospira* in the nitrification process at a rough range of ammonium in eutrophic environments.

## 4 Discussion

The predominance of comammox *Nitrospira* in oligotrophic environments has been studied well, while their contribution to nitrification in eutrophic environments has been poorly understood (Smith and Schindler 2009; Williams and Crutzen 2010; Deegan et al. 2012). Understanding the response of nitrifiers, especially the newly discovered comammox *Nitrospira* to eutrophic conditions, is particularly important for N removal and sustainable aquaculture as the presence of excessive nutrients in ecosystems such as aquaculture is a common issue. In this study, we found that the comammox *Nitrospira* community had higher α-diversity than that of AOB and AOA as expected. Surprisingly, we found that the abundance of comammox *Nitrospira* is equivalent to that of AOB and comammox *Nitrospira* was more sensitive to aquaculture environmental variations than other nitrifiers (AOA and AOB). They played a critical role in the interaction networks of the nitrifiers and the nitrification process in the aquaculture ecosystem and laboratory experiments of mixed-culturing nitrifiers. These results generally support our hypothesis that comammox *Nitrospira* play a major role in the nitrification process in eutrophic aquaculture ecosystems.

The abundance and diversity of comammox *Nitrospira*, AOA, AOB, and NOB responded to nutrient addition differently. In our study, we detected a high abundance of comammox *Nitrospira* which was equivalent to AOB and followed by AOA. The abundance of comammox *Nitrospira* was negatively affected by higher nutrient addition but was still at the same magnitude as AOB and positively correlated to the C/N ratio, which indicated their crucial role in the nitrification process in eutrophic aquaculture ponds. Our results were contrasting with the previous findings which showed the abundance of comammox *Nitrospira* was much lower than that of AOA or AOB in slight eutrophic agricultural soils (Guo et al., 2017; Shi et al., 2018, 2020; Wang et al., 2020; Xu et al., 2020). Their observations could be explained by the difference in substrate affinity among nitrifiers (Prosser and Nicol, 2012; Hu and He, 2017; Kits et al., 2017; Wang et al., 2020; Yue et al., 2022); specifically, comammox *Nitrospira inopinata* have the highest affinity for ammonia (0.65 μM), followed by most AOA (>5.7 μM) and most AOB (>1,000 μM). Comammox *Nitrospira* were generally considered to have a lower growth rate than AOB (Costa et al., 2006; Hu and He, 2017), while the substrate concentration here was between the ammonia affinity of comammox *Nitrospira* and AOB where they were at similar growth rate (Yang et al., 2022). However, eutrophic aquatic environments, such as wastewater treatment plants and coastal aquaculture filter systems, and agricultural soils with higher C/N ratios were shown to harbor a high abundance of comammox *Nitrospira*, which was consistent with our study (Foesel et al., 2008; Annavajhala et al., 2018; Pan et al., 2018; Li et al., 2019; Zheng et al., 2019). The ammonium concentration of our study was 40–70 μM, which was much higher than the affinity of those reported for *N. inopinata*. The high diversity and abundance of comammox *Nitrospira* in these eutrophic aquatic environments may be due to the metabolic versatility of these comammox *Nitrospira* species (Spasov et al., 2020; Xu et al., 2020; Vijayan et al., 2021). In addition, their higher α-diversity indices than canonical ammonium-oxidizers in this study hinted that besides the subtract range, environmental factors, metabolic versatility, growth yields, and biofilm formation capabilities could also affect the diversity and abundance of these newly discovered nitrifiers (Lawson and Lucker, 2018; Spasov et al., 2020). Moreover, we found the proportion of comammox *Nitrospira* clade A significantly had a greater abundance and it was increased along with nutrient addition. These results were consistent with previous findings that clade A was suggested to have a wider distribution, higher diversity, and greater abundance than clade B (Fowler et al., 2018; Shi et al., 2018; Xia et al., 2018; Xu et al., 2020; Zhu et al., 2022) due to their lower ammonia affinity of clade A for ammonia transportation (Koch et al., 2019). These results together suggested the niche heterogeneity and metabolic flexibility of comammox *Nitrospira* may guide diverse niches.

The unabsorbed N of fish feed is largely converted to ammonium through ammonification (Hargreaves, 1998; Hu et al., 2012), which not only increased the N-related parameters but also altered other parameters, thus affecting nitrification activity and nitrifying community (Magill et al., 2000; Kraft et al., 2014; Chen et al., 2016; He et al., 2018, 2020). In this study, we found that AOA and AOB were significantly correlated with several environmental variables including pH, temperature, and salinity. However, comammox *Nitrospira* and NOB communities were sensitive to most of the environmental changes, the changes in N-related parameters could explain more variation in their communities. These results were partly consistent with previous studies that pH, temperature, salinity, and ammonium availability shaped the composition of nitrifying communities especially AOA and AOB communities in agricultural soils and coastal ecosystems (Guo et al., 2017; Xia et al., 2018; Xu et al., 2020; Zhao et al., 2021; Yue et al., 2022). It was reported that N addition altered ammonium availability by reducing pH and increasing substrate concentrations (Hallin et al., 2009; Shi et al., 2018). Furthermore, in this study, increasing temperature and DO only increased the richness of comammox *Nitrospira*, which confirmed that other environmental variables affect the diversity of this newly discovered nitrifier besides ammonium. Moreover, nitrate also positively correlated with the comammox richness while negatively correlated with NOB richness, which hinted the important role of comammox in nitrification and their higher sensitivity to the nitrifying reaction products compared to canonical nitrite oxidizer. In addition to the effect of environmental changes on microbial community assembly, these co-existed microbial species interacting with each other and forming complex microbial community networks played a profound role in various ecosystem services (Barberán et al., 2012; Tu et al., 2020; Yue et al., 2022). In this study, we found that high fish feeding decreased the complexity of nitrifying communities. This result was consistent with an investigation of nitrifying microbial communities’ response to long-term fertilization of inorganic fertilization, not organic fertilization (Banerjee et al., 2016; Yue et al., 2022). It suggested nutrient addition altered the resource-related co-occurrence of microorganisms. Co-occurrence relationships also lie in metabolic connections among microbial groups. For example, co-metabolic interactions between ammonia oxidizers and NOB have been observed in co-culture experiments (Yang et al., 2022) and genome-based exploration (Pachiadaki et al., 2017). Moreover, we found a high proportion of positive interactions among nitrifiers in the fish ponds. Additionally, the interspecific interactions among nitrifiers decreased under higher nutrient input, which may result from the enough N for nitrifiers caused by the nutrient addition (ammonium inhibition) that weakens the co-metabolic interactions among nitrifiers (Yang et al., 2022).

Keystone species hold together the complex microbial interactions in the ecosystem and were suggested as the drivers of microbial structure and functioning (Banerjee et al., 2018). The shift of keystone species caused by environmental changes might alter the function of the entire ecosystem (Banerjee et al., 2018; Herren and McMahon, 2018; Yang et al., 2021). We found that keystone nodes were dramatically different between the SF and LF ponds. NOB served as keystones to maintain nitrification in the SF ponds, which was consistent with previous studies showing that *Nitrospira* NOB were the dominant and keystone species in the Pearl River estuary (Hou et al., 2018), agricultural soils, and soil microcosms (Xu et al., 2020; Xun et al. 2021). While the keystones shifted to comammox *Nitrospira* with higher fish feeding in the LF ponds, implying in addition to driving the nitrification process, comammox *Nitrospira* also contributed to the N removal process as the core connector to maintain the stability and functioning of nitrifying communities in eutrophic aquaculture habitats.

Due to the importance of nitrifiers in N cycling and N removal in various environments, their relative contribution to nitrification especially the newly discovered comammox *Nitrospira* in eutrophic conditions has drawn attention from researchers. It was well known that comammox *Nitrospira* were the dominant species that contributed most to nitrification in oligotrophic environments (Palomo et al., 2016; Roots et al., 2019). However, their contributions to nitrification in eutrophic environments were poorly understood. It was still controversial whether AOA or AOB contributed more to the nitrification process although it has been studied for decades (Jia and Conrad, 2009; Guo et al., 2017; Wang et al., 2021). The newly discovered comammox *Nitrospira* which joined the chaos complicated their contribution to the nitrification process. In our study, we used simplified mix-culturing experiments of nitrifiers to show that comammox *Nitrospira* contributed most to ammonium oxidation under a relatively eutrophic ammonium concentration (0.2 mM) and their contributions decreased significantly with the increase of ammonium supply. This result confirmed that comammox *Nitrospira* could outcompete AOB at a comparable ammonium concentration (Roots et al., 2019), hinting at their importance in less-eutrophic environments. Our result was not consistent with previous findings in eutrophic agriculture soil where AOB were the main contributor to nitrification (Jia and Conrad, 2009; Prosser and Nicol, 2012; Prosser et al., 2020). This may result from the limitations of our mixed nitrifying communities in representing complex ecosystems. We also found that close linkages between nitrification potential and the abundance of comammox *Nitrospira*, diversity of AOB and comammox *Nitrospira*, keystone species of NOB, and comammox *Nitrospira* explained a large proportion of nitrate variation in the fish ponds. These results consistently emphasized the important role of comammox *Nitrospira* in less-eutrophic coastal aquaculture.

## 5 Conclusion

In summary, this study showed that aquaculture activity altered the diversity, richness, abundance, and interactions of nitrifying communities. Unexpected abundant comammox *Nitrospira* were observed in the aquaculture ecosystem, and they were more sensitive to aquaculture environmental changes compared to canonical nitrifiers. Comammox *Nitrospira* species were keystones in shaping the nitrifying communities and played an important role in nitrification potential. This study fills the gap of comammox *Nitrospira* in the coastal eutrophic environment and advances our understanding of their contribution to nitrification. Future studies may focus on understanding the role of comammox *Nitrospira* in nitrifying communities, their contribution to the nitrification process, and underlying mechanisms using synthetic nitrifying communities, RNA-Seq, and ^15^N isotope tracer.

## Data availability statement

The datasets presented in this study can be found in online repositories. The names of the repository/repositories and accession number(s) can be found at: https://www.ncbi.nlm.nih.gov/bioproject/PRJNA834749.

## Author contributions

XY: Conceptualization, Data curation, Formal analysis, Methodology, Software, Validation, Visualization, Writing – original draft, Writing – review & editing. YW: Methodology, Software, Writing – review & editing. LS: Resources, Supervision, Writing – review & editing. HG: Investigation, Methodology, Software, Writing – review & editing. FL: Investigation, Methodology, Software, Writing – review & editing. JD: Investigation, Methodology, Software, Writing – review & editing. JZ: Data curation, Investigation, Writing – review & editing. CW: Resources, Writing – review & editing. ZH: Resources, Writing – review & editing. MX: Supervision, Writing – review & editing. FFL: Writing – review & editing. XZ: Data curation, Formal analysis, Funding acquisition, Investigation, Project administration, Software, Supervision, Writing – review & editing. BW: Funding acquisition, Project administration, Software, Supervision, Writing – review & editing.

## Footnotes

^1^ http://fungene.cme.msu.edu/

^2^ http://www.bioinformatics.babra-ham.ac.uk/projects/fastqc/

^3^ https://multiqc.info/

^4^ http://ieg2.ou.edu/MENA/

^5^ https://github.com/gephi/gephi/

^6^ https://github.com/EricArcher/rfPermute
