# Supplementary material for: Unraveling the important role of comammox Nitrospira to nitrification in the coastal aquaculture system
Source: Front Microbiol. 2024 Apr 23;15:1355859. doi: 10.3389/fmicb.2024.1355859 (PMC11075664; doi:10.3389/fmicb.2024.1355859)
Supplement: Supplementary file 1 [file Table_1.docx]

**Supporting tables and figures**

**Table S1** Primer pairs for amplicon sequencing and qPCR

**Table S2** Abundances of AOA *amoA*, AOB *amoA*, Comammox *Nitrospira amoA*, *Nitrospira nxrB* and 16S rRNA genes in SF and LF pond sediments

**Table S3** The proportion of comammox *Nitrospira amoA* clade A and clade B in SF and LF pond sediments.

**Table S4** Alpha diversity (indexes of observed zOTUs, Chao1, Shannon, Simpson, and PD_whole_tree) of four functional genes in SF and LF ponds.

**Table S5** Pearson/Spearman correlations between environmental parameters and dominant species (relative abundance>1%) in comammox *Nitrospira* community

**Table S6** Pearson/Spearman correlations between environmental parameters and dominant species (relative abundance>1%) in NOB community

**Table S7** Pearson/Spearman correlations between environmental parameters and dominant species (relative abundance>1%) in AOA community

**Table S8** Pearson/Spearman correlations between environmental parameters and dominant species (relative abundance>1%) in AOB community

**Table S9** Topological properties of co-occurrence networks in different aquaculture ponds

**Table S10** Relative abundance of keystones in SF and LF ponds. Data were represented as mean±SEM (n=18). Different letters showed statistical differences (*P*<0.05)

**Figure S1** Beta diversity of four types of nitrifiers

**Figure S2** The linear regression analysis between the observed zOTUs number and environmental variables

**Figure S3** Co-occurrence networks of nitrifiers in different modules in aquaculture ponds

**Table S1** Primer pairs for amplicon sequencing and qPCR

| Detection | Primer Name | 5’-3’ | Reference |
| --- | --- | --- | --- |
| Comammox* | Ntsp-amoA 162F | GGATTTCTGGNTSGATTGGA | (Fowler *et al.*, 2018) |
|  | Ntsp-amoA 359R | WAGTTNGACCACCASTACCA |  |
| AOA | Arch-amoA26F | GACTACATMTTCTAYACWGAYTGGGC | (Park *et al.*, 2008) |
|  | Arch-amoA417R | GGKGTCATRTATGGWGGYAAYGTTGG |  |
| AOA (qPCR) | Arch-amoAF | STAATGGTCTGGCTTAGACG | (Francis *et al.*, 2005) |
|  | Arch-amoAR | GCGGCCATCCATCTGTATGT |  |
| AOB* | amoA-1F | CGGGGTTTCTACTGGTGGT | (Rotthauwe *et al.*, 1997) |
|  | amoA-2R | CCCCCTCGGGAAAGCCTTCTTC |  |
| NOB* | 169f | TACATGTGGTGGAACA | (Pester *et al.*, 2014) |
|  | 638R | CGGTTCTGGTCRATCA |  |

*Primer pairs for amplicon sequencing and qPCR.

**Table S2** Abundances of AOA *amoA*, AOB *amoA*, Comammox *Nitrospira amoA*, *Nitrospira nxrB* and 16S rRNA genes in SF and LF pond sediments. Data were represented as mean±SD (n=18). Different capital letters mean a statistical significance (*P*<0.05) between SF and LF pond sediments based on T-test; different small letters mean a statistical significance (*P*<0.05) among different functional genes within the same fish size pond sediments based on one-way ANOVA.

| Genes | SF | LF |
| --- | --- | --- |
|  | (copies/g wet sediment) | |
| Com *amoA* | (2.18±0.23)×10^7Bb^ | (1.37±0.21)×10^7Ab^ |
| AOA *amoA* | (2.54±0.45)×10^6a^ | (2.69±0.34)×10^6a^ |
| AOB *amoA* | (2.31±0.48)×10^7b^ | (2.30±0.18)×10^7c^ |
| *Nitrospira nxrB* | (1.69±0.50)×10^8c^ | (2.24±0.86)×10^8d^ |
| 16S rRNA | (1.41±0.07)×10^10d^ | (1.47±0.08)×10^10e^ |
| Com/ammonia-oxidizers | 39.44±2.10% | 32.23±2.74% |
| Nitrifiers/16S | 1.77±0.49% | 1.63±0.40% |
| Com/16S | 0.17±0.03%^A^ | 0.092±0.01%^B^ |

**Table S3** The proportion of comammox *Nitrospira amoA* clade A and clade B in SF and LF pond sediments. zOTUs of comammox *Nitrospira amoA* were assigned as clade A or B according to the phylogenetic tree. Data were represented as mean±SD(n=18). Different capital letters mean a statistical significance (*P*<0.05) between SF and LF pond sediments; different small letters mean a statistical significance (*P*<0.05) within the same pond sediments.

| comammox *Nitrospira amoA* | SF ponds | LF ponds |
| --- | --- | --- |
| clade A | 66.05%±23.35%^Aa^ | 89.41%±3.87%^Ba^ |
| clade B | 33.95%±23.35%^Ab^ | 10.59%±3.87%^Bb^ |

**Table S4** Alpha diversity (indexes of observed zOTU, Chao1, Shannon, Simpson, and PD_whole_tree) of four functional genes in SF and LF ponds. Data were represented as mean±SD (n=18). Different capital letters mean a statistical significance (*P*<0.05) between SF and LF pond sediments based on T-test; different small letters mean a statistical significance (*P*<0.05) among different functional genes based on one-way ANOVA.

| Alpha diversity | comammox *amoA* | | AOA *amoA* | | AOB *amoA* | | *Nitrospira nxrB* | |
| --- | --- | --- | --- | --- | --- | --- | --- | --- |
|  | SF | LF | SF | LF | SF | LF | SF | LF |
| Obs. zOTU | 137±18^Ab^ | 147±12^Bc^ | 61±24^a^ | 70±24^b^ | 46±5^Ba^ | 37±6^Aa^ | 606±87^c^ | 582±85^d^ |
| Chao1 | 156±18^b^ | 167±16^c^ | 70±25^a^ | 79±27^b^ | 48±7^Ba^ | 40±7^Aa^ | 806±119^c^ | 742±109^d^ |
| Shannon | 4.39±0.67^b^ | 4.68±0.38^c^ | 3.58±0.50^Aa^ | 4.12±0.53^Bb^ | 3.45±0.29^Ba^ | 3.04±0.19^Aa^ | 6.87±0.40^c^ | 6.90±0.25^d^ |
| Simpson | 0.87±0.11^a^ | 0.92±0.03^b^ | 0.84±0.05^Aa^ | 0.90±0.06^Bb^ | 0.86±0.03^Ba^ | 0.83±0.02^Aa^ | 0.97±0.02^b^ | 0.97±0.01^c^ |
| PD_whole_tree | 43.5±8.9^Ac^ | 54.5±6.7^Bc^ | 1.77±0.41^Aa^ | 2.17±0.31^Ba^ | 1.05±0.19^Ba^ | 0.89±0.20^Aa^ | 23.1±3.0^Ab^ | 28.3±4.0^Bb^ |

**Table S5** Pearson/Spearman correlations between environmental parameters and dominant species (relative abundance>1%) in comammox *Nitrospira* community. Only Pearson/Spearman correlation with significance (False discovery rate (q), adjusted p_value<0.05) were shown in different color gamut, blue means negative correlation, while orange means positive correlations.

| Variables | Water | | | | | | | | | | | | | | | | | | | Sediments | | | | | | |
| --- | --- | --- | --- | --- | --- | --- | --- | --- | --- | --- | --- | --- | --- | --- | --- | --- | --- | --- | --- | --- | --- | --- | --- | --- | --- | --- |
|  | Temp | pH | DO | Sal | Tran | TSS | POC | DOC | TOC | NH_4_^+^ | NO_2_^-^ | NO_3_^-^ | TIN | TON | TN | PO_4_^3-^ | TOP | TP | Chla | TC | TN | TS | TP | ES | AVS | TOC |
| zOTU_2 | 0.360 | -0.281 | -0.012 | -0.082 | -0.381 | 0.474 | -0.649 | -0.579 | -0.708 | -0.083 | 0.305 | 0.488 | 0.466 | -0.236 | 0.365 | -0.557 | -0.295 | -0.454 | -0.583 | 0.222 | 0.155 | -0.171 | 0.556 | -0.462 | -0.587 | 0.360 |
| zOTU_1 | -0.472 | 0.395 | 0.179 | -0.534 | 0.592 | -0.629 | 0.470 | 0.439 | 0.554 | -0.302 | -0.249 | -0.615 | -0.616 | 0.371 | -0.555 | 0.467 | 0.050 | 0.227 | 0.388 | -0.278 | -0.228 | 0.214 | -0.363 | 0.458 | 0.480 | -0.472 |
| zOTU_3 | 0.362 | -0.343 | -0.121 | -0.170 | -0.492 | 0.548 | -0.423 | -0.542 | -0.620 | -0.112 | 0.455 | 0.628 | 0.597 | -0.212 | 0.535 | -0.696 | -0.298 | -0.561 | -0.546 | 0.214 | 0.149 | -0.265 | 0.565 | -0.472 | -0.636 | 0.362 |
| zOTU_4 | 0.354 | -0.272 | -0.046 | -0.009 | -0.315 | 0.412 | -0.600 | -0.501 | -0.613 | 0.055 | 0.300 | 0.513 | 0.495 | -0.231 | 0.398 | -0.545 | -0.173 | -0.354 | -0.544 | 0.312 | 0.247 | -0.103 | 0.577 | -0.468 | -0.541 | 0.354 |
| zOTU_5 | -0.077 | -0.091 | -0.182 | 0.512 | -0.057 | 0.028 | -0.117 | 0.216 | 0.110 | 0.163 | -0.044 | 0.062 | 0.033 | -0.207 | 0.015 | 0.187 | 0.170 | 0.165 | 0.190 | -0.033 | -0.004 | 0.096 | -0.298 | 0.147 | 0.020 | -0.077 |
| zOTU_6 | -0.051 | 0.063 | -0.041 | 0.596 | 0.018 | -0.118 | 0.057 | 0.362 | 0.299 | 0.124 | -0.240 | -0.067 | -0.112 | -0.122 | -0.133 | 0.181 | 0.243 | 0.215 | 0.288 | 0.037 | 0.079 | 0.242 | -0.330 | 0.248 | 0.171 | -0.051 |
| zOTU_7 | -0.206 | 0.223 | 0.107 | 0.491 | 0.366 | -0.409 | 0.186 | 0.549 | 0.528 | 0.156 | -0.391 | -0.482 | -0.446 | 0.003 | -0.476 | 0.557 | 0.289 | 0.526 | 0.497 | -0.111 | -0.052 | 0.430 | -0.474 | 0.578 | 0.584 | -0.206 |
| zOTU_9 | 0.429 | -0.239 | -0.010 | -0.101 | -0.413 | 0.468 | -0.534 | -0.494 | -0.622 | -0.015 | 0.322 | 0.527 | 0.471 | -0.252 | 0.388 | -0.599 | -0.318 | -0.507 | -0.630 | 0.239 | 0.200 | -0.209 | 0.516 | -0.498 | -0.632 | 0.429 |
| zOTU_8 | 0.565 | -0.468 | -0.213 | 0.302 | -0.643 | 0.732 | -0.394 | -0.649 | -0.720 | 0.298 | 0.502 | 0.708 | 0.722 | -0.428 | 0.627 | -0.592 | -0.325 | -0.449 | -0.518 | 0.172 | 0.125 | -0.401 | 0.436 | -0.596 | -0.730 | 0.565 |
| zOTU_10 | 0.133 | 0.185 | 0.170 | 0.762 | 0.130 | -0.086 | -0.016 | 0.371 | 0.275 | 0.282 | -0.379 | -0.275 | -0.235 | -0.168 | -0.301 | 0.423 | 0.222 | 0.479 | 0.261 | 0.096 | 0.134 | 0.315 | -0.286 | 0.349 | 0.315 | 0.133 |
| zOTU_11 | 0.107 | 0.362 | 0.331 | 0.470 | 0.189 | -0.190 | -0.049 | 0.430 | 0.347 | 0.063 | -0.453 | -0.464 | -0.397 | 0.201 | -0.375 | 0.518 | 0.262 | 0.538 | 0.237 | 0.166 | 0.215 | 0.343 | -0.268 | 0.357 | 0.444 | 0.107 |
| zOTU_14 | -0.408 | -0.221 | -0.255 | 0.006 | 0.251 | -0.102 | -0.076 | 0.029 | -0.024 | 0.218 | 0.177 | 0.120 | 0.084 | -0.309 | -0.050 | 0.042 | -0.137 | -0.048 | 0.009 | -0.358 | -0.322 | -0.007 | -0.355 | 0.060 | 0.066 | -0.408 |
| zOTU_12 | -0.405 | 0.220 | -0.086 | -0.085 | 0.424 | -0.468 | 0.304 | 0.500 | 0.551 | -0.010 | -0.183 | -0.385 | -0.372 | 0.024 | -0.442 | 0.331 | 0.156 | 0.219 | 0.411 | -0.105 | -0.068 | 0.275 | -0.400 | 0.401 | 0.352 | -0.405 |
| zOTU_18 | 0.027 | -0.396 | -0.332 | 0.302 | -0.235 | 0.265 | -0.354 | -0.104 | -0.218 | 0.393 | 0.310 | 0.391 | 0.423 | -0.479 | 0.303 | -0.297 | 0.013 | -0.179 | -0.267 | -0.199 | -0.196 | -0.271 | -0.133 | -0.260 | -0.383 | 0.027 |
| zOTU_15 | 0.162 | -0.533 | -0.445 | -0.232 | -0.520 | 0.554 | -0.216 | -0.596 | -0.572 | -0.095 | 0.604 | 0.622 | 0.591 | -0.297 | 0.543 | -0.729 | -0.499 | -0.790 | -0.271 | 0.061 | 0.014 | -0.243 | 0.383 | -0.318 | -0.534 | 0.162 |
| zOTU_17 | -0.010 | 0.399 | 0.290 | 0.522 | 0.289 | -0.402 | 0.148 | 0.566 | 0.515 | 0.118 | -0.462 | -0.448 | -0.419 | 0.057 | -0.449 | 0.522 | 0.288 | 0.533 | 0.321 | -0.012 | 0.022 | 0.328 | -0.480 | 0.329 | 0.357 | -0.010 |
| zOTU_16 | 0.289 | -0.329 | -0.141 | -0.189 | -0.369 | 0.430 | -0.416 | -0.644 | -0.689 | -0.033 | 0.402 | 0.475 | 0.446 | -0.171 | 0.396 | -0.526 | -0.306 | -0.464 | -0.430 | -0.036 | -0.091 | -0.415 | 0.362 | -0.606 | -0.672 | 0.289 |
| zOTU_13 | -0.605 | 0.192 | -0.057 | -0.377 | 0.421 | -0.465 | 0.493 | 0.475 | 0.613 | -0.315 | -0.183 | -0.385 | -0.417 | 0.426 | -0.235 | 0.272 | 0.157 | 0.168 | 0.480 | 0.043 | 0.060 | 0.376 | -0.117 | 0.543 | 0.535 | -0.605 |
| zOTU_19 | -0.098 | 0.315 | 0.225 | 0.412 | 0.267 | -0.306 | 0.037 | 0.560 | 0.443 | -0.049 | -0.403 | -0.524 | -0.500 | 0.170 | -0.489 | 0.480 | 0.265 | 0.497 | 0.410 | -0.033 | 0.014 | 0.373 | -0.368 | 0.496 | 0.606 | -0.098 |
| zOTU_20 | 0.235 | -0.239 | -0.035 | -0.159 | -0.293 | 0.420 | -0.470 | -0.470 | -0.553 | -0.046 | 0.331 | 0.398 | 0.393 | -0.177 | 0.300 | -0.339 | -0.229 | -0.371 | -0.462 | 0.053 | -0.023 | -0.233 | 0.326 | -0.345 | -0.625 | 0.235 |

**Table S6** Pearson/Spearman correlations between environmental parameters and dominant species (relative abundance>1%) in NOB community. Only Pearson/Spearman correlation with significance (False discovery rate (q), adjusted p_value<0.05) were shown in different color gamut, blue means negative correlation, while orange means positive correlations.

| Variables | Water | | | | | | | | | | | | | | | | | | | Sediments | | | | | | |
| --- | --- | --- | --- | --- | --- | --- | --- | --- | --- | --- | --- | --- | --- | --- | --- | --- | --- | --- | --- | --- | --- | --- | --- | --- | --- | --- |
|  | Temp | pH | DO | Sal | Tran | TSS | POC | DOC | TOC | NH_4_^+^ | NO_2_^-^ | NO_3_^-^ | TIN | TON | TN | PO_4_^3-^ | TOP | TP | Chla | TC | TN | TS | TP | ES | AVS | TOC |
| zOTU_1 | 0.294 | -0.504 | -0.379 | 0.199 | -0.576 | 0.647 | -0.314 | -0.568 | -0.646 | 0.192 | 0.522 | 0.662 | 0.664 | -0.465 | 0.556 | -0.513 | -0.301 | -0.425 | -0.346 | -0.048 | -0.069 | -0.438 | 0.242 | -0.544 | -0.753 | -0.003 |
| zOTU_2 | -0.032 | -0.163 | -0.137 | -0.609 | -0.131 | 0.119 | -0.093 | -0.244 | -0.205 | -0.295 | 0.294 | 0.293 | 0.271 | 0.145 | 0.336 | -0.355 | -0.351 | -0.474 | -0.361 | 0.052 | 0.008 | -0.172 | 0.259 | -0.112 | -0.236 | -0.003 |
| zOTU_3 | -0.425 | 0.330 | 0.139 | -0.305 | 0.619 | -0.620 | 0.196 | 0.432 | 0.470 | -0.108 | -0.298 | -0.574 | -0.578 | 0.160 | -0.605 | 0.502 | 0.124 | 0.309 | 0.399 | -0.408 | -0.385 | 0.158 | -0.561 | 0.472 | 0.465 | -0.447 |
| zOTU_4 | 0.303 | -0.282 | -0.170 | 0.155 | -0.346 | 0.397 | -0.550 | -0.423 | -0.531 | 0.171 | 0.192 | 0.486 | 0.447 | -0.312 | 0.358 | -0.486 | -0.115 | -0.366 | -0.455 | 0.258 | 0.159 | -0.127 | 0.390 | -0.457 | -0.481 | 0.344 |
| zOTU_8 | -0.335 | 0.467 | 0.264 | 0.196 | 0.506 | -0.645 | 0.529 | 0.694 | 0.787 | -0.083 | -0.527 | -0.700 | -0.691 | 0.322 | -0.594 | 0.686 | 0.401 | 0.654 | 0.742 | 0.035 | 0.091 | 0.570 | -0.376 | 0.679 | 0.792 | -0.024 |
| zOTU_7 | -0.256 | -0.450 | -0.423 | -0.300 | 0.055 | 0.099 | -0.076 | -0.091 | -0.113 | 0.438 | 0.522 | 0.223 | 0.250 | -0.450 | 0.097 | -0.217 | 0.016 | -0.146 | 0.044 | -0.520 | -0.516 | -0.366 | -0.320 | -0.098 | -0.110 | -0.528 |
| zOTU_5 | 0.511 | -0.476 | -0.219 | 0.097 | -0.675 | 0.759 | -0.487 | -0.703 | -0.793 | 0.138 | 0.467 | 0.760 | 0.742 | -0.370 | 0.660 | -0.727 | -0.404 | -0.688 | -0.675 | 0.065 | -0.005 | -0.497 | 0.403 | -0.629 | -0.755 | 0.148 |
| zOTU_9 | -0.381 | -0.432 | -0.477 | -0.455 | -0.024 | 0.179 | 0.106 | -0.028 | -0.037 | 0.088 | 0.553 | 0.249 | 0.224 | -0.138 | 0.228 | -0.235 | -0.091 | -0.236 | 0.100 | -0.360 | -0.346 | -0.341 | -0.118 | -0.118 | -0.033 | -0.387 |
| zOTU_11 | -0.317 | 0.597 | 0.417 | -0.098 | 0.635 | -0.750 | 0.357 | 0.660 | 0.737 | -0.185 | -0.656 | -0.810 | -0.796 | 0.381 | -0.760 | 0.661 | 0.257 | 0.559 | 0.456 | 0.158 | 0.206 | 0.693 | -0.229 | 0.677 | 0.743 | 0.093 |
| zOTU_10 | 0.380 | -0.303 | -0.013 | 0.176 | -0.240 | 0.394 | -0.652 | -0.497 | -0.646 | 0.244 | 0.185 | 0.418 | 0.454 | -0.366 | 0.253 | -0.365 | -0.084 | -0.300 | -0.579 | -0.150 | -0.253 | -0.443 | 0.036 | -0.511 | -0.578 | -0.065 |
| zOTU_13 | -0.364 | 0.562 | 0.369 | -0.009 | 0.625 | -0.713 | 0.382 | 0.681 | 0.750 | -0.239 | -0.644 | -0.742 | -0.747 | 0.439 | -0.660 | 0.669 | 0.277 | 0.527 | 0.489 | 0.109 | 0.137 | 0.646 | -0.308 | 0.661 | 0.786 | 0.052 |
| zOTU_15 | -0.401 | 0.574 | 0.386 | -0.122 | 0.681 | -0.749 | 0.256 | 0.698 | 0.724 | -0.274 | -0.656 | -0.836 | -0.828 | 0.412 | -0.785 | 0.671 | 0.279 | 0.557 | 0.448 | 0.034 | 0.085 | 0.654 | -0.299 | 0.759 | 0.803 | -0.026 |
| zOTU_12 | 0.414 | -0.320 | -0.141 | 0.224 | -0.494 | 0.574 | -0.444 | -0.643 | -0.734 | 0.060 | 0.301 | 0.685 | 0.616 | -0.346 | 0.554 | -0.543 | -0.261 | -0.479 | -0.558 | 0.130 | 0.097 | -0.407 | 0.413 | -0.641 | -0.643 | 0.212 |
| zOTU_14 | -0.434 | -0.451 | -0.488 | -0.262 | 0.011 | 0.159 | 0.110 | -0.012 | -0.043 | 0.017 | 0.461 | 0.332 | 0.293 | -0.143 | 0.288 | -0.202 | -0.201 | -0.303 | 0.046 | -0.564 | -0.557 | -0.466 | -0.324 | -0.150 | -0.125 | -0.566 |
| zOTU_16 | 0.286 | -0.483 | -0.311 | 0.147 | -0.471 | 0.581 | -0.549 | -0.458 | -0.602 | 0.282 | 0.409 | 0.578 | 0.587 | -0.446 | 0.461 | -0.609 | -0.115 | -0.391 | -0.475 | 0.210 | 0.156 | -0.203 | 0.427 | -0.522 | -0.529 | 0.314 |
| zOTU_17 | -0.425 | -0.438 | -0.495 | -0.399 | 0.033 | 0.134 | 0.103 | -0.055 | -0.072 | 0.071 | 0.523 | 0.307 | 0.259 | -0.239 | 0.227 | -0.266 | -0.184 | -0.400 | 0.019 | -0.506 | -0.512 | -0.435 | -0.275 | -0.197 | -0.130 | -0.526 |
| zOTU_18 | 0.263 | -0.399 | -0.262 | 0.151 | -0.439 | 0.478 | -0.526 | -0.353 | -0.480 | 0.166 | 0.278 | 0.550 | 0.521 | -0.379 | 0.418 | -0.576 | -0.092 | -0.428 | -0.409 | 0.137 | 0.062 | -0.177 | 0.301 | -0.410 | -0.451 | 0.232 |

**Table S7** Pearson/Spearman correlations between environmental parameters and dominant species (relative abundance>1%) in AOA community. Only Pearson/Spearman correlation with significance (False discovery rate (q), adjusted p_value<0.05) were shown in different color gamut, blue means negative correlation, while orange means positive correlations.

| Variables | Water | | | | | | | | | | | | | | | | | | | Sediments | | | | | | |
| --- | --- | --- | --- | --- | --- | --- | --- | --- | --- | --- | --- | --- | --- | --- | --- | --- | --- | --- | --- | --- | --- | --- | --- | --- | --- | --- |
|  | Temp | pH | DO | Sal | Tran | TSS | POC | DOC | TOC | NH_4_^+^ | NO_2_^-^ | NO_3_^-^ | TIN | TON | TN | PO_4_^3-^ | TOP | TP | Chla | TC | TN | TS | TP | ES | AVS | TOC |
| zOTU_1 | 0.144 | 0.217 | 0.235 | 0.531 | 0.016 | 0.029 | -0.003 | 0.328 | 0.192 | 0.220 | -0.174 | -0.242 | -0.223 | 0.027 | -0.224 | 0.390 | 0.262 | 0.485 | 0.212 | -0.037 | -0.020 | 0.063 | -0.263 | 0.148 | 0.118 | -0.043 |
| zOTU_2 | -0.382 | -0.103 | -0.186 | -0.627 | 0.251 | -0.174 | -0.064 | -0.243 | -0.155 | -0.199 | 0.073 | 0.101 | 0.059 | -0.027 | -0.013 | -0.248 | -0.137 | -0.370 | -0.278 | -0.239 | -0.266 | -0.045 | -0.062 | 0.008 | -0.166 | -0.257 |
| zOTU_3 | 0.052 | -0.281 | -0.192 | -0.014 | -0.339 | 0.331 | -0.283 | -0.490 | -0.520 | -0.005 | 0.257 | 0.355 | 0.316 | -0.146 | 0.300 | -0.407 | 0.011 | -0.270 | -0.206 | -0.171 | -0.196 | -0.369 | 0.098 | -0.420 | -0.322 | -0.101 |
| zOTU_9 | 0.004 | 0.060 | 0.087 | 0.359 | -0.035 | 0.136 | 0.101 | 0.265 | 0.154 | 0.154 | -0.050 | -0.173 | -0.136 | 0.068 | -0.113 | 0.271 | 0.361 | 0.441 | 0.184 | -0.102 | -0.079 | -0.017 | -0.214 | 0.072 | 0.135 | -0.112 |
| zOTU_4 | 0.147 | 0.108 | 0.164 | 0.408 | 0.024 | 0.082 | -0.109 | 0.256 | 0.094 | 0.297 | -0.089 | -0.188 | -0.121 | -0.090 | -0.161 | 0.248 | 0.174 | 0.328 | 0.041 | -0.108 | -0.087 | -0.044 | -0.226 | 0.068 | 0.099 | -0.111 |
| zOTU_7 | 0.276 | -0.002 | 0.014 | 0.458 | -0.348 | 0.216 | 0.129 | 0.199 | 0.132 | 0.269 | 0.073 | 0.001 | 0.017 | 0.108 | 0.143 | 0.043 | 0.357 | 0.322 | 0.235 | 0.100 | 0.110 | -0.125 | 0.007 | -0.083 | -0.008 | 0.076 |
| zOTU_5 | 0.274 | -0.076 | 0.041 | 0.225 | -0.309 | 0.307 | -0.433 | -0.324 | -0.439 | -0.094 | -0.006 | 0.215 | 0.236 | -0.098 | 0.212 | -0.246 | -0.151 | -0.133 | -0.325 | 0.192 | 0.186 | -0.069 | 0.325 | -0.280 | -0.197 | 0.259 |
| zOTU_6 | -0.124 | -0.236 | -0.257 | -0.103 | -0.059 | 0.060 | -0.196 | -0.292 | -0.268 | 0.179 | 0.250 | 0.273 | 0.295 | -0.287 | 0.161 | -0.239 | 0.026 | -0.229 | -0.141 | -0.425 | -0.440 | -0.342 | -0.355 | -0.164 | -0.260 | -0.414 |
| zOTU_8 | -0.119 | -0.277 | -0.322 | -0.224 | -0.155 | 0.053 | 0.168 | -0.266 | -0.135 | 0.032 | 0.356 | 0.283 | 0.273 | -0.125 | 0.272 | -0.394 | -0.052 | -0.304 | -0.024 | -0.154 | -0.121 | -0.291 | 0.139 | -0.271 | -0.165 | -0.114 |
| zOTU_10 | -0.534 | -0.154 | -0.338 | -0.535 | 0.271 | -0.252 | 0.125 | -0.061 | 0.048 | -0.141 | 0.255 | -0.027 | -0.079 | -0.031 | -0.114 | -0.111 | -0.052 | -0.211 | 0.237 | -0.165 | -0.132 | 0.096 | -0.005 | 0.116 | -0.005 | -0.156 |
| zOTU_13 | -0.267 | 0.089 | -0.030 | -0.592 | 0.214 | -0.228 | 0.133 | -0.140 | -0.037 | -0.433 | 0.094 | -0.098 | -0.180 | 0.204 | -0.111 | -0.105 | -0.252 | -0.359 | 0.115 | -0.335 | -0.314 | -0.119 | -0.179 | 0.108 | -0.075 | -0.343 |
| zOTU_12 | 0.251 | -0.255 | -0.080 | -0.009 | -0.395 | 0.441 | -0.199 | -0.486 | -0.508 | 0.018 | 0.254 | 0.509 | 0.482 | -0.164 | 0.485 | -0.524 | -0.139 | -0.398 | -0.555 | 0.136 | 0.128 | -0.278 | 0.426 | -0.549 | -0.468 | 0.197 |
| zOTU_11 | 0.204 | -0.141 | -0.061 | -0.356 | -0.250 | 0.138 | -0.116 | -0.345 | -0.271 | -0.040 | 0.280 | 0.272 | 0.272 | -0.105 | 0.257 | -0.427 | -0.193 | -0.351 | -0.220 | 0.230 | 0.229 | -0.067 | 0.459 | -0.252 | -0.259 | 0.237 |
| zOTU_14 | 0.204 | -0.141 | -0.061 | -0.356 | -0.250 | 0.138 | -0.116 | -0.345 | -0.271 | -0.040 | 0.280 | 0.272 | 0.272 | -0.105 | 0.257 | -0.427 | -0.193 | -0.351 | -0.220 | 0.230 | 0.229 | -0.067 | 0.459 | -0.252 | -0.259 | 0.237 |
| zOTU_15 | 0.045 | -0.269 | -0.107 | -0.073 | -0.194 | 0.300 | -0.388 | -0.336 | -0.416 | -0.077 | 0.192 | 0.318 | 0.292 | -0.010 | 0.305 | -0.332 | -0.114 | -0.268 | -0.314 | -0.135 | -0.159 | -0.279 | 0.036 | -0.347 | -0.352 | -0.076 |
| zOTU_17 | 0.118 | 0.038 | 0.147 | 0.312 | -0.051 | 0.105 | -0.068 | 0.254 | 0.128 | 0.245 | -0.078 | -0.142 | -0.074 | -0.021 | -0.077 | 0.191 | 0.232 | 0.321 | -0.023 | -0.082 | -0.096 | 0.036 | -0.288 | 0.145 | 0.124 | -0.114 |
| zOTU_16 | 0.224 | 0.114 | 0.090 | 0.586 | -0.168 | 0.096 | -0.011 | 0.051 | 0.038 | 0.126 | -0.146 | -0.053 | 0.002 | -0.015 | 0.066 | 0.117 | 0.365 | 0.345 | 0.186 | 0.234 | 0.263 | 0.035 | 0.001 | -0.104 | 0.009 | 0.259 |
| zOTU_18 | 0.078 | -0.010 | 0.002 | 0.015 | -0.165 | 0.245 | -0.234 | -0.248 | -0.302 | -0.223 | -0.024 | 0.106 | 0.087 | 0.085 | 0.153 | -0.084 | -0.086 | -0.059 | -0.157 | 0.424 | 0.446 | 0.108 | 0.543 | -0.203 | -0.058 | 0.434 |
| zOTU_21 | 0.015 | 0.170 | 0.207 | 0.347 | 0.072 | 0.011 | 0.014 | 0.387 | 0.251 | 0.068 | -0.213 | -0.288 | -0.268 | 0.145 | -0.229 | 0.340 | 0.370 | 0.461 | 0.172 | 0.023 | 0.045 | 0.155 | -0.174 | 0.198 | 0.216 | 0.005 |

**Table S8** Pearson/Spearman correlations between environmental parameters and dominant species (relative abundance>1%) in AOB community. Only Pearson/Spearman correlation with significance (False discovery rate (q), adjusted p_value<0.05) were shown in different color gamut, blue means negative correlation, while orange means positive correlations.

| Variables | Water | | | | | | | | | | | | | | | | | | | Sediments | | | | | | |
| --- | --- | --- | --- | --- | --- | --- | --- | --- | --- | --- | --- | --- | --- | --- | --- | --- | --- | --- | --- | --- | --- | --- | --- | --- | --- | --- |
|  | Temp | pH | DO | Sal | Tran | TSS | POC | DOC | TOC | NH_4_^+^ | NO_2_^-^ | NO_3_^-^ | TIN | TON | TN | PO_4_^3-^ | TOP | TP | Chla | TC | TN | TS | TP | ES | AVS | TOC |
| zOTU_1 | 0.359 | 0.051 | 0.074 | 0.408 | -0.301 | 0.137 | -0.119 | -0.069 | -0.114 | 0.022 | -0.148 | 0.085 | 0.160 | -0.109 | 0.082 | -0.178 | -0.038 | -0.019 | -0.040 | 0.344 | 0.282 | 0.112 | 0.212 | -0.059 | -0.026 | 0.357 |
| zOTU_2 | -0.006 | -0.304 | -0.311 | -0.190 | -0.190 | 0.355 | -0.238 | -0.542 | -0.578 | 0.059 | 0.481 | 0.478 | 0.348 | -0.279 | 0.367 | -0.323 | -0.284 | -0.381 | -0.326 | -0.231 | -0.273 | -0.483 | 0.058 | -0.528 | -0.520 | -0.197 |
| zOTU_3 | 0.316 | 0.018 | 0.073 | 0.539 | -0.192 | 0.243 | -0.214 | -0.130 | -0.209 | 0.013 | -0.105 | 0.154 | 0.173 | -0.173 | 0.129 | -0.070 | 0.125 | -0.015 | -0.068 | 0.146 | 0.124 | -0.061 | 0.204 | -0.156 | -0.251 | 0.221 |
| zOTU_4 | -0.508 | -0.128 | -0.316 | -0.386 | 0.130 | -0.193 | 0.308 | 0.167 | 0.276 | 0.011 | 0.267 | -0.067 | -0.097 | 0.153 | -0.011 | -0.017 | 0.372 | 0.121 | 0.475 | -0.165 | -0.102 | 0.018 | -0.074 | 0.123 | 0.117 | -0.203 |
| zOTU_5 | -0.074 | -0.238 | -0.100 | -0.218 | 0.014 | 0.158 | -0.173 | -0.081 | -0.111 | 0.110 | 0.220 | 0.141 | 0.068 | -0.204 | 0.067 | -0.026 | -0.490 | -0.266 | -0.359 | -0.205 | -0.189 | -0.012 | -0.237 | 0.040 | 0.021 | -0.198 |
| zOTU_6 | -0.041 | -0.185 | -0.118 | -0.387 | -0.109 | 0.059 | -0.104 | -0.115 | -0.064 | -0.269 | 0.075 | 0.083 | 0.067 | 0.177 | 0.120 | -0.258 | -0.120 | -0.334 | -0.195 | 0.163 | 0.124 | 0.092 | 0.311 | 0.061 | -0.051 | 0.158 |
| zOTU_7 | -0.411 | -0.188 | -0.278 | -0.369 | 0.201 | -0.191 | 0.311 | 0.290 | 0.371 | 0.116 | 0.151 | -0.066 | -0.096 | 0.118 | -0.027 | -0.037 | 0.188 | 0.012 | 0.219 | 0.027 | 0.093 | 0.138 | -0.067 | 0.155 | 0.347 | -0.071 |
| zOTU_8 | -0.089 | -0.059 | -0.090 | -0.343 | 0.120 | -0.044 | -0.011 | -0.196 | -0.208 | -0.096 | 0.187 | -0.015 | -0.041 | -0.144 | -0.105 | -0.089 | -0.310 | -0.277 | -0.017 | -0.476 | -0.472 | -0.409 | -0.237 | -0.286 | -0.117 | -0.467 |

**Table S9** Topological properties of co-occurrence networks in different aquaculture ponds

| Network Indexes | SF | LF |
| --- | --- | --- |
| Total nodes | 476 | 289 |
| Total links | 1429 | 348 |
| R square of power-law | 0.808 | 0.83 |
| Average degree (avgK) | 6.004 | 2.408 |
| Average clustering coefficient (avgCC) | 0.423 | 0.285 |
| Average path distance (GD) | 9.397 | 3.446 |
| Geodesic efficiency (E) | 0.153 | 0.42 |
| Harmonic geodesic distance (HD) | 6.53 | 2.381 |
| Maximal degree | 24 | 9 |
| Nodes with max degree | nxrB_38 | com_130; com_134; nxrB_113 |
| Centralization of degree (CD) | 0.038 | 0.023 |
| Maximal betweenness | 28687.225 | 244.833 |
| Nodes with max betweenness | nxrB_112 | nxrB_99 |
| Centralization of betweenness (CB) | 0.242 | 0.006 |
| Maximal stress centrality | 3958576 | 415 |
| Nodes with max stress centrality | nxrB_97 | nxrB_99 |
| Centralization of stress centrality (CS) | 33.713 | 0.009 |
| Maximal eigenvector centrality | 0.267 | 0.436 |
| Nodes with max eigenvector centrality | nxrB_38 | nxrB_113 |
| Centralization of eigenvector centrality (CE) | 0.256 | 0.424 |
| Density (D) | 0.013 | 0.008 |
| Reciprocity | 1 | 1 |
| Transitivity (Trans) | 0.55 | 0.409 |
| Connectedness (Con) | 0.75 | 0.051 |
| Efficiency | 0.986 | 0.887 |
| Hierarchy | 0 | 0 |
| Lubness | 1 | 1 |

**Table S10** Relative abundance of keystones in SF and LF ponds. Data were represented as mean ± SEM (n=18). Different letters showed statistical differences (*P*<0.05).

| zOTUs | Nitrifiers | | Relative abundance (%) | |
| --- | --- | --- | --- | --- |
|  |  |  | SF | LF |
| zOTU_94 | *Nitrospira nxrB* | Uncultured *Nitrospira* | 0.40±0.07^b^ | 0.02±0.01^a^ |
| zOTU_247 | *Nitrospira nxrB* | Others (New *Nitrospira*) | 0.08±0.02 | 0.04±0.01 |
| zOTU_1316 | *Nitrospira nxrB*  comammox clade A | *Nitrospira* sp. ST-bin5 | 0.00±0.00 | 0.00±0.00 |
| zOTU_29 | comammox *Nitrospira amoA* cladeB | Uncultured *Nitrospira* | 0.08±0.03^a^ | 1.07±0.29^b^ |
| zOTU_57 | comammox *Nitrospira amoA* cladeA | Uncultured *Nitrospira* | 0.23±0.06 | 0.20±0.03 |
| zOTU_103 | comammox *Nitrospira* *amoA* cladeA | Others (New *Nitrospira*) | 0.06±0.02 | 0.05±0.03 |
| zOTU_134 | comammox *Nitrospira amoA* cladeA | Others (New *Nitrospira*) | 0.02±0.07 | 0.07±0.06 |


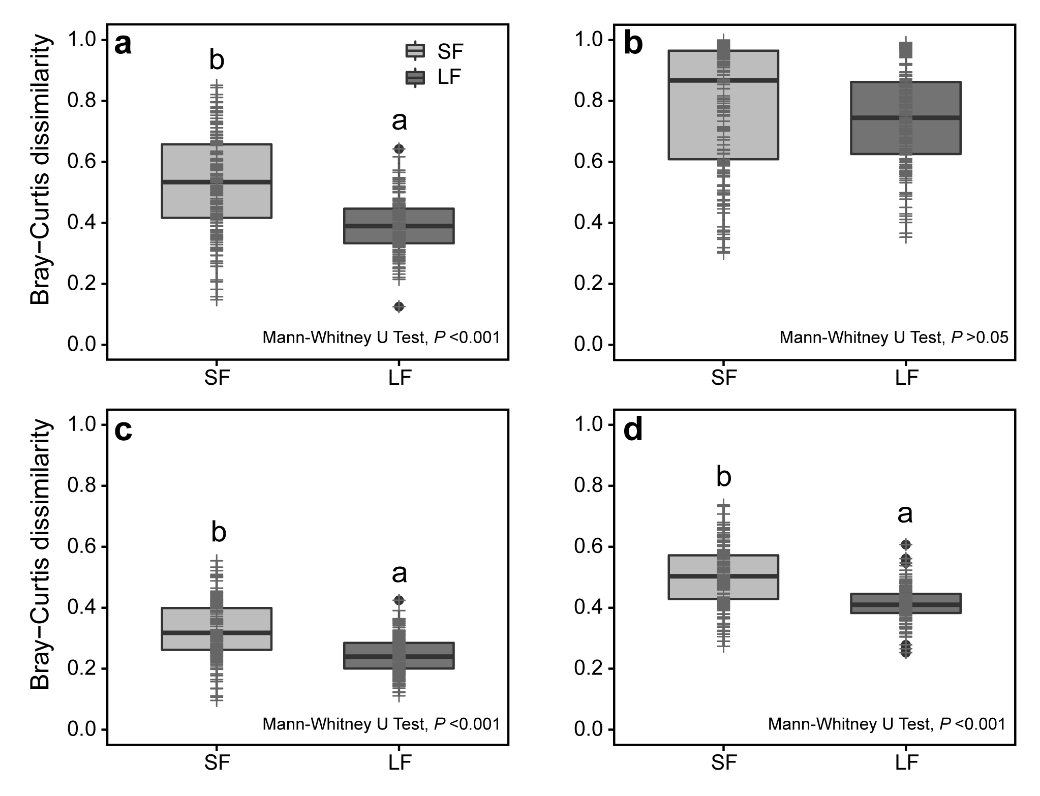


**Figure S1** Beta diversity of four types of nitrifiers (a) comammox *Nitrospira amoA*, (b) AOA *amoA*, (c) AOB *amoA* and (d) *Nitrospira nxrB* in SF and LF pond sediments were estimated based on a Bray-Curtis distance matrix. Different letters showed statistical differences (*P*<0.001, Mann-Whitney U-test).


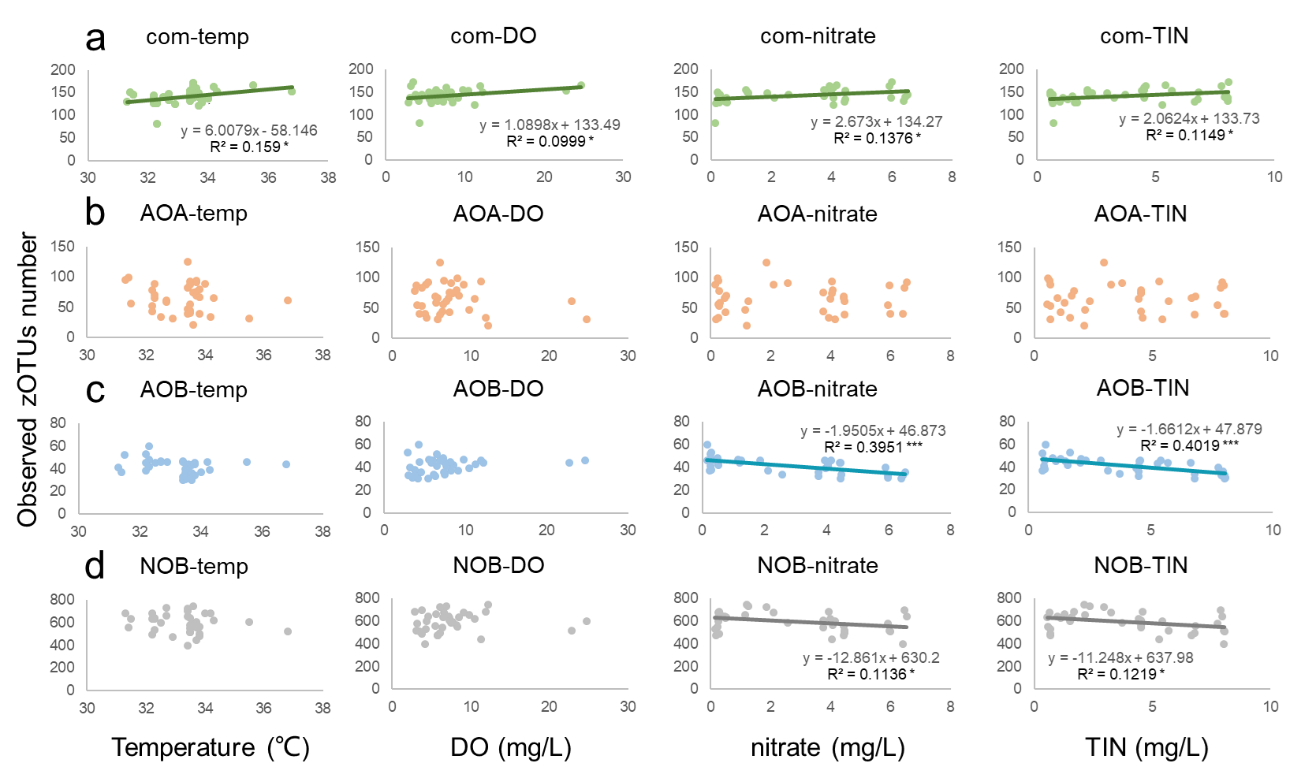


**Figure S2** The linear regression analysis between the observed zOTUs number of nitrifiers including (a) comammox Nitrospira, (b) AOA, (c) AOB, (D) NOB and environmental variables (Temperature, DO, nitrate and TIN). Only liner regression with significance showed in solid line (* means *P*<0.05, ** *P*<0.01, ****P*<0.001).


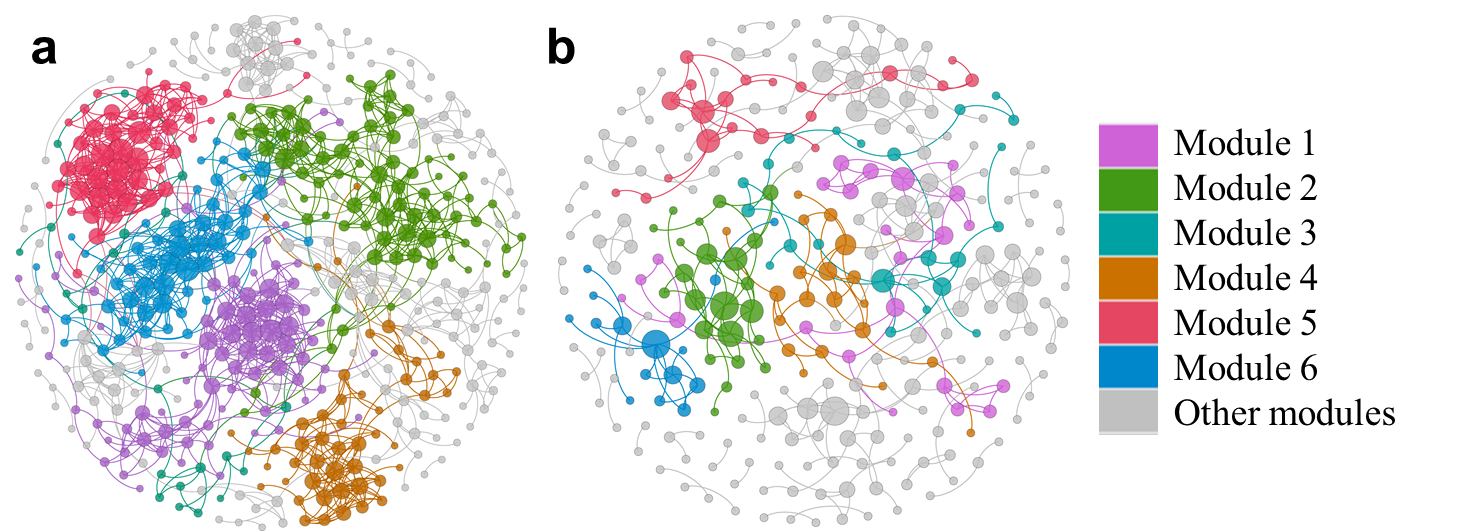


**Figure S3** Co-occurrence networks of nitrifiers in different modules in aquaculture ponds, (a) SF ponds; (b) LF ponds.

**Reference**

Fowler, S. J., Palomo, A., Dechesne, A., Mines, P. D., and Smets, B. F. (2018). Comammox *Nitrospira* are abundant ammonia oxidizers in diverse groundwater-fed rapid sand filter communities. *Environ. Microbiol.* 20, 1002-1015.

Francis, C. A., Roberts, K. J., Beman, J. M., Santoro, A. E., and Oakley, B. B. (2005). Ubiquity and diversity of ammonia-oxidizing archaea in water columns and sediments of the ocean. *Proc. Natl. Acad. Sci. U. S. A.* 102, 14683-14688.

Park, S.-J., Park, B.-J., and Rhee, S.-K. (2008). Comparative analysis of archaeal 16S rRNA and *amoA* genes to estimate the abundance and diversity of ammonia-oxidizing archaea in marine sediments. *Extremophiles* 12, 605-615.

Pester, M., Maixner, F., Berry, D., Rattei, T., Koch, H., Lucker, S., Nowka, B., Richter, A., Spieck, E., Lebedeva, E., Loy, A., Wagner, M., and Daims, H. (2014). *NxrB* encoding the beta subunit of nitrite oxidoreductase as functional and phylogenetic marker for nitrite-oxidizing *Nitrospira*. *Environ. Microbiol.* 16, 3055-3071.

Rotthauwe, J.-H., Witzel, K.-P., and Liesack, W. (1997). The ammonia monooxygenase structural gene *amoA* as a functional marker: Molecular fine-scale analysis of natural ammonia-oxidizing populations. *Appl. Environ. Microbiol.* 63, 4704-4712.
